# Robust predictive framework for diabetes classification using optimized machine learning on imbalanced datasets

**DOI:** 10.3389/frai.2024.1499530

**Published:** 2025-01-07

**Authors:** Inam Abousaber, Haitham F. Abdallah, Hany El-Ghaish

**Affiliations:** ^1^Department of Information Technology, Faculty of Computers and Information Technology, University of Tabuk, Tabuk, Saudi Arabia; ^2^Department of Electronics and Electrical Communication, Higher Institute of Engineering and Technology, Kafr El Sheikh, Egypt; ^3^Department of Computer and Automatic Control, Faculty of Engineering, Tanta University, Tanta, Egypt

**Keywords:** diabetes detection, imbalance handling methods, imbalanced datasets, machine learning, statistical analysis

## Abstract

**Introduction:**

Diabetes prediction using clinical datasets is crucial for medical data analysis. However, class imbalances, where non-diabetic cases dominate, can significantly affect machine learning model performance, leading to biased predictions and reduced generalization.

**Methods:**

A novel predictive framework employing cutting-edge machine learning algorithms and advanced imbalance handling techniques was developed. The framework integrates feature engineering and resampling strategies to enhance predictive accuracy.

**Results:**

Rigorous testing was conducted on three datasets—PIMA, Diabetes Dataset 2019, and BIT_2019—demonstrating the robustness and adaptability of the methodology across varying data environments.

**Discussion:**

The experimental results highlight the critical role of model selection and imbalance mitigation in achieving reliable and generalizable diabetes predictions. This study offers significant contributions to medical informatics by proposing a robust data-driven framework that addresses class imbalance challenges, thereby advancing diabetes prediction accuracy.

## 1 Introduction

Diabetes is a chronic disease that has reached epidemic proportions globally, affecting ~537 million adults as of 2021, with projections indicating a rise to 783 million by 2045 (Saeedi et al., 2019). Characterized by the body's inability to produce or effectively use insulin, diabetes leads to elevated blood glucose levels, which, if not managed, can result in severe complications such as cardiovascular disease, kidney failure, blindness, and lower limb amputations (Demir et al., 2021). These complications diminish the quality of life for millions of people and significantly increase healthcare costs, placing a considerable burden on healthcare systems worldwide (Tomic et al., 2022).

Early detection of diabetes is critical for timely intervention, which can significantly reduce the risk of these complications and improve patient outcomes (Jones et al., 2021). By diagnosing diabetes early, patients can receive appropriate treatment, make necessary lifestyle adjustments, and closely monitor their condition, preventing or delaying the onset of severe complications. Early intervention is significant for stopping the progression from prediabetes to type 2 diabetes, which affects a substantial proportion of at-risk individuals (Kaur et al., 2020).

Traditional methods of diabetes detection, often reliant on fasting blood glucose levels or HbA1c measurements, have limitations, including the potential for late diagnosis and the requirement for clinical visits (Park et al., 2020). For many patients, particularly those in underserved or remote areas, access to regular healthcare services is limited, resulting in delayed diagnoses and treatment. Moreover, these traditional diagnostic methods may not be sensitive enough to detect diabetes at its earliest stages, when intervention can be most effective, Ortiz-Martínez et al. (2022) highlighting the urgent requirement for easy-to-access, precise and earlier diagnostic solutions that can be deployed in a range of healthcare environments including either primary care or telemedicine.

However, in the past few years, machine learning (ML) has emerged as a promising option, facilitating the deployment of predictive models which allow the analysis of large-scale patient data, thus predicting accurately and at an early stage who is at risk of diabetes (Johnson, 2024). These models are game-changers that could transform how we care for diabetes patients with earlier diagnoses, customized treatment pathways and better patient management. Yet their performance is often hindered by the imbalance characteristic of medical datasets. Diabetic cases are way lower in number than non-diabetics in such datasets, which may make the models biased toward predicting the majority class and also not that great when detecting the minority class (Johnson, 2024).

Patients can end up with a bad deal in this imbalance. If a predictive model cannot predict those in the presumably early stages of diabetes or high susceptibility to developing diabetes, and these patients do not receive sufficient early interventions to impede the deteriorating process. This means that they are more likely to have the worst possible outcomes of unmanaged diabetes, which could have been prevented if it had been presented early and appropriately intervened (Gao et al., 2020). Therefore, addressing the imbalance in diabetic datasets is not just a technical challenge but a critical issue that directly impacts patient health and outcomes.

Dealing with this disparity necessitates some clever approaches, both in preprocessing data and choosing our model. Approaches such as SMOTE (Synthetic Minority Over-sampling Technique), ADASYN (Adaptive Synthetic Sampling), and its variants are used to synthetically create samples of the minority class, making the dataset balanced (Brandt and Lanzén, 2021). Also, ensemble methods to reduce the complexity of decision trees and advanced algorithms such as Random Forests, Gradient Boosting Trees, or Support Vector Machines (SVM) have been experimented with to classify data more effectively in imbalanced classes (Zhou et al., 2023). These approaches enhance the accuracy of predictions and ensure that the models are sensitive to the critical minority class, thereby improving the chances of early detection and intervention for at-risk patients.

This paper builds upon the foundation of work presented in Abousaber (2024). This study aims to address these challenges by systematically evaluating various machine learning techniques across three distinct datasets: PIMA (Nelson et al., 2021), Diabetic Dataset 2019 (Tigga and Garg, 2020), and BIT_2019 (Zhang et al., 2024). The datasets have their characteristics and challenges, making them a rich set of benchmarks for testing the generalizability of our methods. After undergoing advanced preprocessing techniques and dealing with the imbalance, we prepared data for model training. Various machine learning models were then trained, and their performance was extensively assessed, especially in correctly classifying the minority diabetic class.

Overall, this study aims to fill this gap between the increasing necessity for early diabetic detection and the difficulties faced with imbalanced datasets. We sought to create a comprehensive framework using a large set of machine learning algorithms and advanced preprocessing techniques, which led to improved predictive performance and generalized models across diverse populations for mortality. These insights could have marked ramifications in the world of medical diagnostics, providing better instruments for early-stage detection of diabetes and hence leading to better patient prognosis and quality of life.

The following contributions highlight this research's novelty in advancing methodologies within medical informatics and addressing key challenges in diabetes prediction:

1. **Integrated Framework for Enhanced Modeling:** This study presents a framework that combines feature engineering with resampling techniques (SMOTE, ADASYN, Borderline-SMOTE) to address class imbalance, enhancing model accuracy and stability.
2. **High Accuracy with Optimized Ensemble Models:** The framework uses optimized ensemble models (Random Forest, XGBoost, and LightGBM) with resampling to achieve balanced, clinically relevant predictions.
3. **Validated Across Diverse Datasets:** Tested on benchmark datasets (PIMA, Diabetes Dataset 2019, BIT_2019), the framework demonstrates consistent effectiveness across varied populations.
4. **Significant Impact on Medical Informatics:** This work addresses class imbalance in diabetes prediction, supporting early detection and aiding clinical decision-making to improve patient outcomes.

The remainder of this paper is organized as follows. Section 2 reviews the existing literature on machine learning approaches for diabetes detection, focusing on the challenges of imbalanced datasets. In Section 3, we introduce the datasets utilized in this study—PIMA, Diabetic Dataset 2019, and BIT_2019—and detail the preprocessing steps, including data cleaning, feature engineering, the techniques employed to handle class imbalance, and the various machine learning models evaluated in this research and the training and hyperparameter tuning strategies implemented to optimize their performance. The overall work methodology is illustrated in Section 4. The experimental results and discussion is included in Section 5. Finally, Section 7 concludes the paper by summarizing the key findings, discussing their implications for diabetic detection, and proposing directions for future research.

## 2 Related work

Machine learning (ML) has become a cornerstone in diabetes detection research, driven by the need for early diagnosis and intervention. Early studies primarily employed simple models like Logistic Regression and Decision Trees due to their interpretability and ease of use. For example, Edlitz and Segal (2022) successfully used Logistic Regression to pinpoint key risk factors, forming a basis for more advanced models. However, these approaches often struggled with the complex, non-linear relationships typical of medical data, particularly in imbalanced datasets where non-diabetic cases outnumber diabetic cases.

Researchers have increasingly turned to ensemble methods such as Random Forests and Gradient Boosting to address these limitations, which combine weak learners to enhance classification. Palimkar et al. (2022) showed that Random Forests can improve classification accuracy by reducing overfitting, while Poria and Jaiswal (2022) highlighted Gradient Boosting's strength in capturing complex data relationships. SVMs have also proven effective in diabetes prediction, with Tan et al. (2023) demonstrating how feature selection with SVMs can significantly improve performance. Meanwhile, Cheng et al. (2020) showcased the potential of deep learning models to automate feature extraction, outperforming traditional methods on large datasets.

A major obstacle in diabetes prediction is the imbalance in datasets, where diabetic cases are far fewer than non-diabetic ones. This can lead to biased models that excel in identifying non-diabetic cases but fail to detect diabetic ones. Resampling techniques like SMOTE (Pears et al., 2014) and ADASYN (Zakariah et al., 2023) have been widely adopted to address this. Aubaidan et al. (2024) showed that SMOTE can improve model recall for minority classes, while ADASYN, as used by Zakariah et al., adjusts sampling based on instance difficulty, further enhancing model sensitivity to diabetic cases.

Combining ensemble methods with resampling techniques has also shown promise. Ganie et al. (2023) developed an ensemble approach incorporating SMOTE to boost sensitivity and classification performance in diabetic datasets. Hazarika and Gupta (2022) introduced Density-Weighted Twin SVM (DWTWSVM), assigning weights to minority class samples to reduce bias, which proved highly effective in handling class imbalance through metrics like F1-score and G-mean.

Despite these advances, systematic comparisons of different imbalance-handling strategies across multiple diabetic datasets remain limited. Our study addresses this gap by evaluating various ML models, including Logistic Regression, Decision Trees, and multiple ensemble techniques, alongside advanced resampling methods across three prominent datasets: PIMA, Diabetes Dataset 2019, and BIT_2019. This approach aims to establish generalizable, effective strategies for diabetes detection.

Interpretability is another crucial aspect in healthcare applications, where model transparency is key for clinical adoption. Chang et al. (2023) underscored this by using interpretable models like Naïve Bayes, Random Forest, and J48 Decision Trees, showing their value in clinical decision-making. Similarly, You and Kang (2020a) demonstrated how SVM and Decision Tree models, combined with correlation analysis, can aid in identifying diabetes risk factors with a clear rationale.

More complex models like artificial neural networks (ANNs) have also been explored. Lakhwani et al. (2020) proposed a three-layer ANN with promising accuracy, while Bhargava et al. (2020) improved KNN accuracy by introducing the Standard Deviation K-Nearest Neighbor (SDKNN). Somwanshi (2021) further validated SVM on the PIMA dataset, showing its effectiveness in clinical prediction with standardized data preprocessing. Additionally, Zhang et al. (2024) developed a non-invasive Back Propagation Neural Network (BPNN) for diabetes diagnosis, achieving notable improvements in accuracy, sensitivity, and specificity through batch normalization.

In recent work, Uddin et al. (2024) demonstrated the effectiveness of a multi-model approach combining Linear Regression, Logistic Regression, KNN, Naïve Bayes, Random Forest, SVM, and Decision Tree, achieving high accuracy across the Diabetes Dataset 2019 and Pima Indian datasets by leveraging SMOTE and other preprocessing techniques. This highlights the necessity of balancing datasets to minimize false negatives and maximize predictive accuracy in clinical applications.

Our study builds on these advancements by systematically comparing ML models and imbalance handling methods across multiple diabetic datasets, establishing a robust framework for accurate, interpretable, and fair diabetes detection. This approach highlights the significance of model selection and preprocessing and sets a foundation for further development of reliable ML models in medical diagnostics.

## 3 Datasets and preprocessing

### 3.1 Datasets

The datasets utilized in this study include the PIMA Indians Diabetes Database (Nelson et al., 2021), the Diabetic Dataset 2019 (Tigga and Garg, 2020), and the BIT_2019 dataset (Zhang et al., 2024). Each dataset presents unique characteristics and challenges, making them suitable for evaluating the robustness of different machine-learning models and preprocessing techniques.

#### 3.1.1 PIMA Indians diabetes database

**Figure 1:**
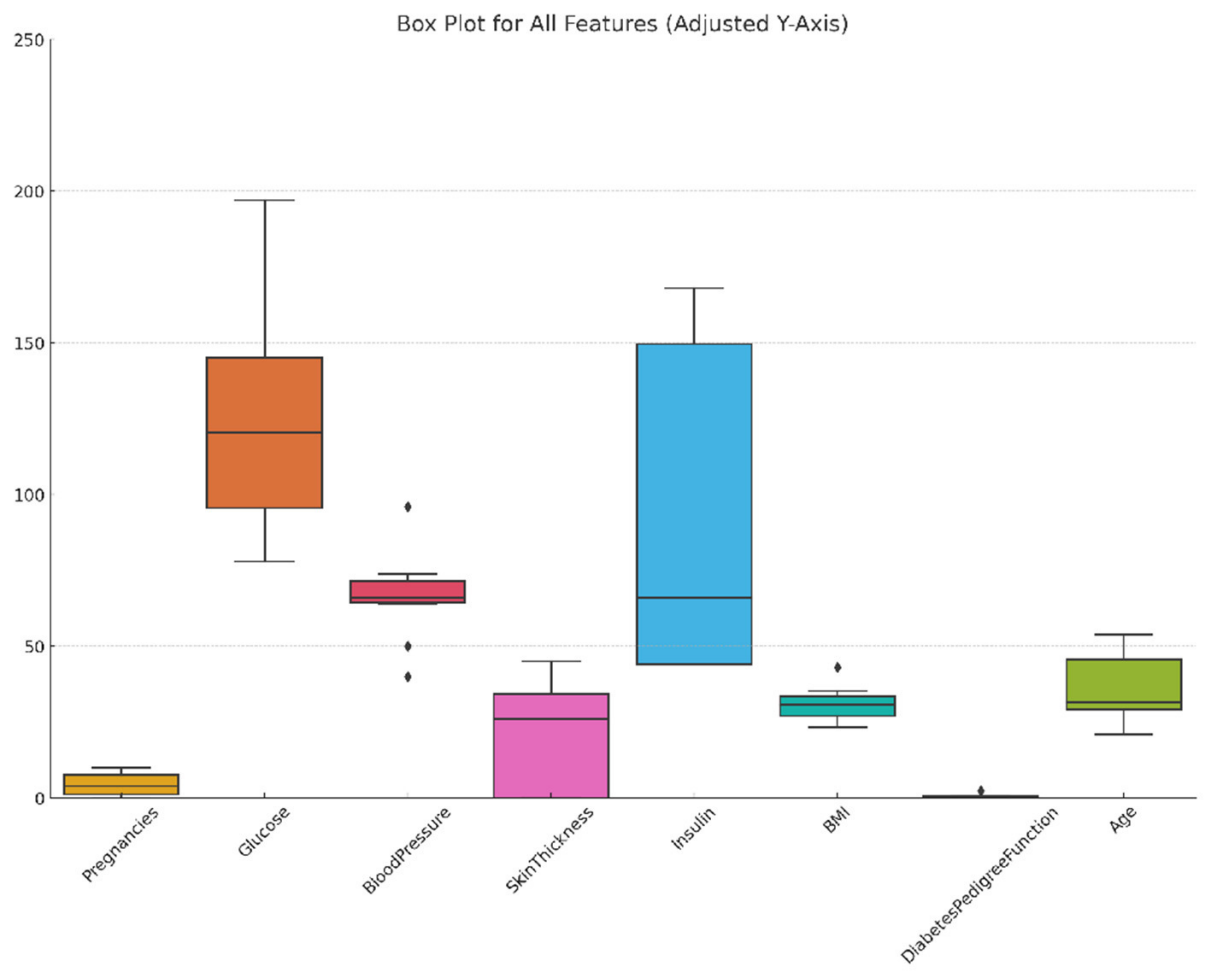
Box plot of selected features in the PIMA dataset showing distribution, variability, and outliers.

The PIMA Indian diabetes dataset, a benchmark dataset, is provided by the National Institute of Diabetes and Digestive and Kidney Diseases (NIDDK) in collaboration with the Applied Physics Laboratory at Johns Hopkins University (Nelson et al., 2021). It comprises 768 observations of female patients of PIMA Indian heritage aged 21 years or older. The dataset includes eight features: number of pregnancies (*Pregnancies*), plasma glucose concentration after 2 hours in an oral glucose tolerance test (*Glucose*), diastolic blood pressure (*BloodPressure*), triceps skinfold thickness (*SkinThickness*), 2-h serum insulin (*Insulin*), body mass index (*BMI*), a diabetes pedigree function reflecting family history (*DiabetesPedigreeFunction*), and the patient's age in years (*Age*). The target variable, *Outcome*, indicates whether the patient has diabetes (1) or not (0). The dataset is imbalanced, with 268 positive cases and 500 negative cases. Figure 1 shows the distribution, variability, and outliers of selected features in the PIMA dataset, providing insights into feature spread and potential anomalies.

#### 3.1.2 Diabetic dataset 2019

**Figure 2:**
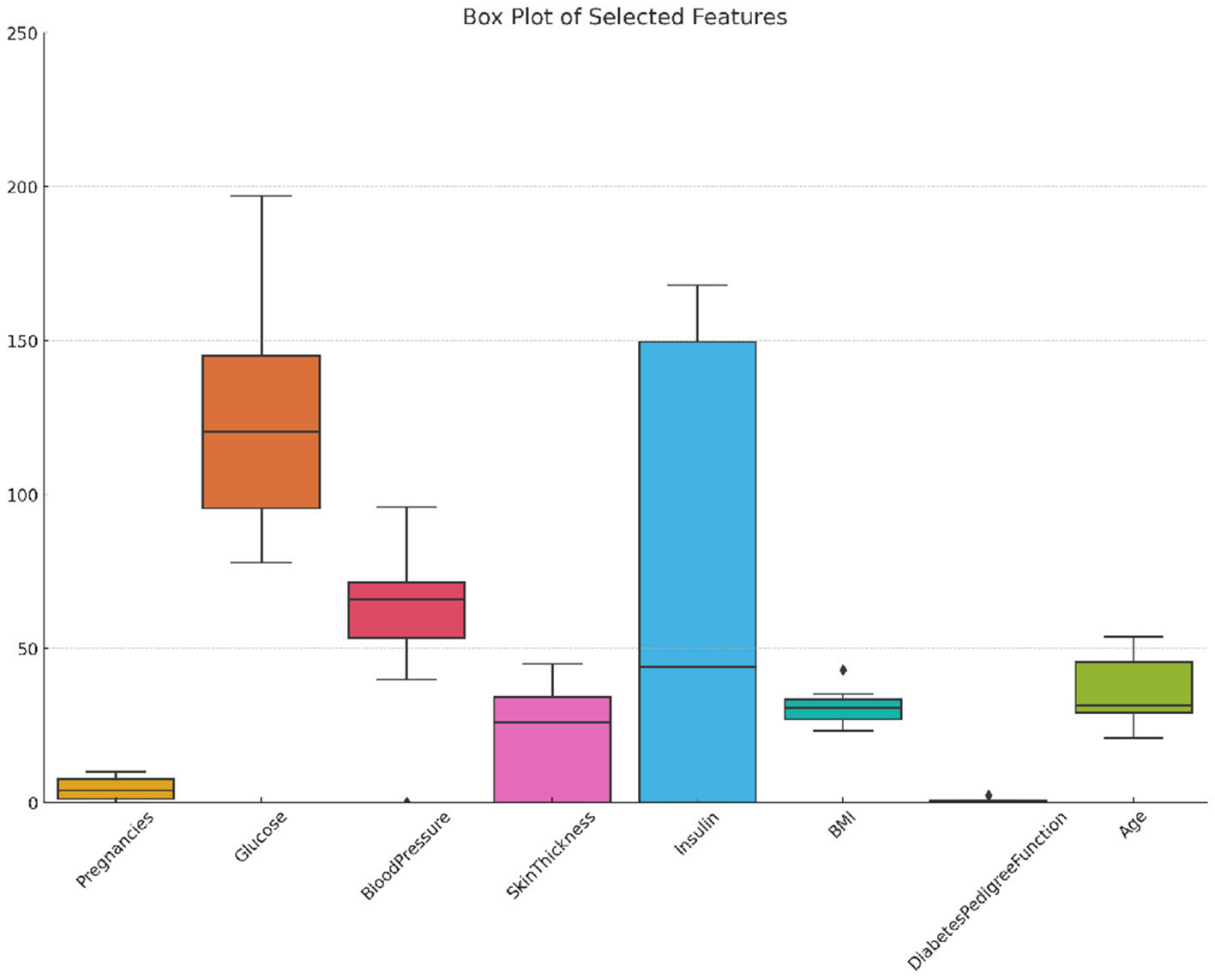
Box plot of selected features in the diabetic 2019 dataset showing distribution, variability, and outliers.

The Diabetic dataset 2019 consists of 520 observations, incorporating more features than the PIMA dataset. It includes traditional medical metrics like the number of pregnancies (*Pregnancies*), plasma glucose concentration (*Glucose*), systolic and diastolic blood pressure levels (*BloodPressure*), body mass index (*BMI*), and a diabetes pedigree function (*DiabetesPedigreeFunction*), along with age categorized into four groups (*Age*) (Tigga and Garg, 2020). This data set also records some lifestyles, including gender, family history of diabetes, smoking status, drinking status, regular use of medication, physical activity level, dietary habits, stress levels, urination frequency, etc, and blood pressure levels. The binary target variable, *Diabetic*, denotes diabetic (1) or non-diabetic (0) status, with an imbalanced distribution of 170 diabetic cases and 350 non-diabetic cases. Figure 2 illustrates the distribution, variability, and outliers of selected features in the Diabetic 2019 dataset, offering a visual summary of feature spread and identifying potential anomalies.

#### 3.1.3 BIT_2019 dataset

**Figure 3:**
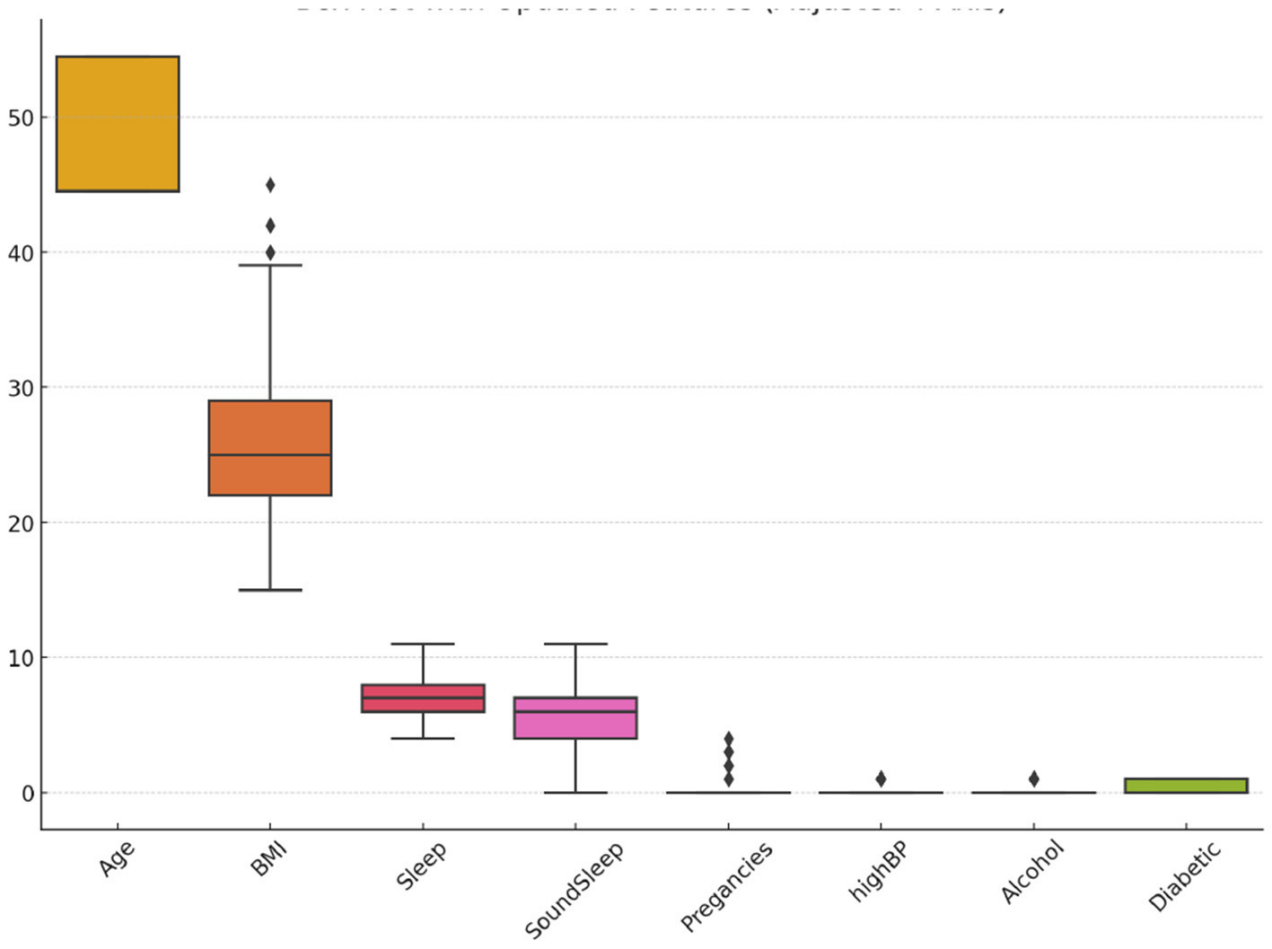
Box plot of selected features in the BIT_2019 dataset showing distribution, variability, and outliers.

The BIT_2019 dataset, collected by the Birla Institute of Technology, Mesra, includes 952 observations. It features a diverse set of attributes, including age (categorized into four groups), gender, family history of diabetes, presence of high blood pressure, physical activity levels, body mass index, smoking and alcohol consumption, hours of sleep and sound sleep, regular medication use, frequency of junk food consumption, stress levels, blood pressure levels, number of pregnancies, prediabetes status, and urination frequency (Zhang et al., 2024). The target variable, *Diabetic*, indicates whether the patient has diabetes (1) or non-diabetic (0), with 320 diabetic cases and 632 non-diabetic cases, highlighting the dataset's class imbalance. Figure 3 displays a box plot analysis of selected features in the BIT_2019 dataset, highlighting the range, central tendency, and presence of outliers to reveal feature distribution characteristics and variability.

As shown in Table 1, all three datasets exhibit significant class imbalance, with the number of non-diabetic samples far exceeding the number of diabetic samples in each case. This imbalance challenges the development of machine learning models, which may become biased toward the majority class. Various resampling techniques and preprocessing steps were applied to ensure the models could accurately identify diabetic cases despite the imbalance.

**Table 1:** Class distribution in the datasets.

**Dataset**	**Total samples**	**Non-diabetic samples**	**Diabetic samples**
PIMA Indians diabetes	768	500	268
Diabetic dataset 2019	945	680	265
BIT_2019	905	642	263

The datasets described in Table 1 are divided with an 80–20 split, where 80% of the samples are used for training, and 20% are reserved for testing. This division aligns with the approach adopted for the PIMA dataset as seen in Rajagopal et al. (2022), Singh and Singh (2020), Kibria et al. (2022), and Chang et al. (2023). Similarly, the diabetic dataset 2019 follows the same 80–20 division as reported by Uddin et al. (2024), and the BIT_2019 dataset is also partitioned into 80% training and 20% testing as stated in Zhang et al. (2024).

### 3.2 Data preprocessing

Effective data preprocessing is crucial for enhancing machine learning models, particularly when handling imbalanced datasets (Karatas et al., 2020; Nelson et al., 2021). The preparation procedures differed among the datasets to accommodate their distinct attributes.

#### 3.2.1 Handling missing values

Different strategies were employed to handle missing data depending on the dataset:

- In the **PIMA dataset**, missing values in features like *Glucose, Insulin*, and *BMI* were replaced with the median of the respective feature, which is particularly effective for addressing missing data in small datasets (Nelson et al., 2021).
- The **Diabetic dataset 2019** required a more extensive approach. Entries with significant missing data were removed while missing values in categorical variables such as *Age* and *Physical Activity* were imputed using the mode, and continuous variables were imputed using the median.
- For the **BIT_2019 dataset**, instances with extensive missing data were removed, and categorical variables were label encoded. Missing values in continuous variables were also imputed using the median to maintain dataset integrity.

#### 3.2.2 Feature engineering and scaling

To enhance the predictive power of the models, polynomial feature expansion was applied across all datasets to capture potential non-linear relationships (Karatas et al., 2020).

**Polynomial feature** expansion generates new features by creating combinations of existing features raised to a specified power and their interactions. Given a dataset with features *x*_1_, *x*_2_, …, *x*_*n*_, a polynomial expansion of degree 2 including the transformations in Equation 7. This expansion allows the model to learn interactions between features that are not linear, improving its ability to capture complex patterns in the data.

**Correlation**: Following polynomial expansion, a **correlation matrix** was computed to examine the relationships between the newly generated features. The Pearson correlation coefficient *r*_*ij*_ between any two features *x*_*i*_ and *x*_*j*_ is defined as:


(1) $$\begin{array}{l} r_{ij}=\frac{\text{cov}\left(x_{i},x_{j}\right)}{\sigma_{x_{i}}\sigma_{x_{j}}} \end{array}$$

where cov(*x*_*i*_, *x*_*j*_) represents the covariance between features *x*_*i*_ and *x*_*j*_, and σ_*x*_*i*__ and σ_*x*_*j*__ are the standard deviations of *x*_*i*_ and *x*_*j*_, respectively.

To prevent multicollinearity features with a correlation coefficient |*r*_*ij*_| > 0.9 were considered highly correlated, and one feature from each highly correlated pair was removed from the dataset. This step helps to ensure that the model does not overfit redundant information and maintains generalizability.

**Scaling**: After feature selection, StandardScaler was applied to normalize the data:


(2) $$\begin{array}{l} \text{Standard Scaling: }x_{i}^{\prime }=\frac{x_{i}-\mu_{i}}{\sigma_{i}} \end{array}$$

where *x*_*i*_ is the original feature value, μ_*i*_ is the mean of the feature, and σ_*i*_ is the standard deviation of the feature. This scaling process ensures that each feature contributes equally to the model's learning process, particularly for distance-based algorithms such as SVM and KNN.

#### 3.2.3 Imbalance handling techniques

Given the inherent class imbalance in the datasets—where non-diabetic cases often outnumber diabetic cases—various resampling techniques were applied to address this issue (Rawat and Mishra, 2022; Sadeghi et al., 2022):

- **ADASYN:** The Adaptive Synthetic Sampling (ADASYN) technique generates synthetic samples for the minority class based on the data distribution. ADASYN focuses on generating more synthetic samples for minority class instances that are harder to learn. The number of synthetic samples generated (*G*) is given by:
(3) $$\begin{array}{l} G=\left(\text{X}\times \Delta \right)\times r_{i}\times h_{i} \end{array}$$
  where *X* is the feature vector, Δ is the difference between feature vectors of a minority and majority class instance, *r*_*i*_ is the ratio of minority to majority instances, and *h*_*i*_ is a random value between 0 and 1 (Alhudhaif, 2021).
- **SMOTE:** The Synthetic Minority Over-sampling Technique (SMOTE) generates synthetic samples by interpolating between existing minority class examples. For a feature vector *x* from the minority class, SMOTE selects one of its nearest neighbors *x*_*nn*_ and generates a synthetic example as follows:
(4) $$\begin{array}{l} \text{Synthetic sample}=x+\left(x_{nn}-x\right)\times \delta \end{array}$$
  where δ is a random number between 0 and 1 (Mansourifar and Shi, 2020).
- **Borderline-SMOTE:** An extension of SMOTE, Borderline-SMOTE focuses on generating synthetic samples for minority class instances near the decision boundary (borderline examples). The synthetic samples are generated by interpolating between borderline examples and their nearest neighbors, ensuring that the decision boundary is better defined. The sample generation follows the same equation as SMOTE but is selectively applied to instances near the decision boundary (Sun et al., 2023).
- **RandomUnderSampler:** This technique involves randomly under-sampling the majority class to achieve balance. While this approach can lead to information loss, it is useful when the majority class is enormous compared to the minority class. This method does not have a complex mathematical basis; it simply reduces the size of the majority class by random selection. The process can be described as:
(5) $$\begin{array}{l} X_{\text{majority}}^{\text{sampled}}\subseteq X_{\text{majority}},\;|X_{\text{majority}}^{\text{sampled}}|=|X_{\text{minority}}| \end{array}$$
  where *X*_majority_ represents the set of all instances belonging to the majority class, and $X_{\text{majority}}^{\text{sampled}}$ is a randomly selected subset such that the size of this subset is equal to the size of the minority class (Saylı and Başarır, 2022).
- **SMOTEENN:** The SMOTEENN technique combines SMOTE with Edited Nearest Neighbors (ENN), which removes samples misclassified by their neighbors after oversampling. This combination allows for synthetic sample generation and noise reduction in the dataset. The synthetic sample generation within SMOTEENN follows the same equation as SMOTE:
(6) $$\begin{array}{l} \text{Synthetic sample}=x+\left(x_{nn}-x\right)\times \delta \end{array}$$
  Where δ is a random number between 0 and 1. The ENN step iteratively removes misclassified examples (Ependi et al., 2023).

### 3.3 Machine learning models

In this study, we employ a diverse set of machine learning models, each selected for its unique strengths and suitability for diabetic detection. From simple, interpretable algorithms to complex state-of-the-art frameworks, our results provide a broad assessment of predictive performance across methodologies.

Various machine learning models and classifiers are learned, starting with **Logistic Regression**. This linear model provides the probability of a given input vector being actual using the logistic function (Zaidi and Al Luhayb, 2023). Additionally, **K-Nearest Neighbors (KNN)** uses non-parametric techniques to classify a sample based on the majority vote of its *k* nearest neighbors (Shi, 2020), and **Decision Trees** create splits in the data by selecting features that maximize information gain (Priyanka and Kumar, 2020). The ensemble method **Random Forest** aggregates predictions from multiple decision trees to enhance robustness (Ignacio et al., 2020). Meanwhile, **Gradient Boosting** sequentially builds models to correct errors from previous models, while **Support Vector Machines (SVM)** seeks the hyperplane that best separates classes (Manoharan et al., 2022).

The study also explores probabilistic models such as **Naive Bayes**, which assumes conditional independence among features (Liu et al., 2024), and **XGBoost**, an optimized gradient boosting framework with regularization to prevent overfitting (Dong et al., 2022). **LightGBM** and **CatBoost** are other gradient-boosting frameworks designed for efficiency, especially when handling large datasets or categorical features (Saleem et al., 2024). **Neural Networks**, particularly Multi-Layer Perceptrons, mimic the brain's architecture and are trained using backpropagation (Qamar and Zardari, 2023). Finally, **Balanced Bagging** addresses class imbalances by combining bagging with sampling techniques, ensuring that both minority and majority classes are adequately represented in the data (Malek et al., 2023).

## 4 Methodology

The primary objective of this study is to develop and evaluate machine learning models for the early detection of diabetes, mainly focusing on the challenges posed by imbalanced datasets (as described in Section 3). The research addresses fundamental questions regarding the most effective models and techniques for diabetic prediction and evaluates the impact of different class imbalance handling methods.

### 4.1 Overview of the approach

**Figure 4:**
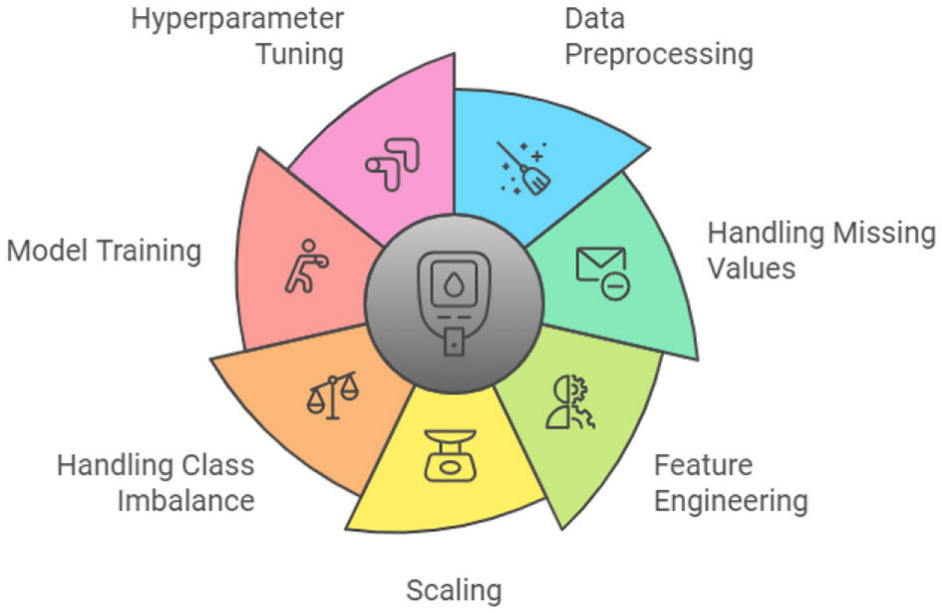
Overview of the methodology steps for diabetic prediction using machine learning.

As Figure 4 illustrates, this study follows a systematic approach. The methodology begins with data collection and preprocessing, followed by feature engineering, scaling and handling class imbalance through various techniques, as discussed in Section 4.2. The prepared data is then used for model selection and training, detailed in Section 4.3, before finally evaluating the models using comprehensive metrics, outlined in Section 4.4.

### 4.2 Data preprocessing

Data preprocessing is critical, ensuring that the input data is high quality and suitable for training the machine learning models. The detailed steps of data preprocessing for each dataset are described in Section 3.

**Handling missing values:** As outlined in Section 3, missing data was managed through median imputation for continuous variables and mode imputation for categorical variables. These methods were particularly effective in maintaining dataset integrity.

#### 4.2.1 Feature engineering and scaling

Feature engineering involved polynomial feature expansion, which was applied to capture non-linear relationships between features.

**Polynomial feature expansion:** Polynomial feature expansion generates new features by raising existing features to a specified degree and creating interaction terms. For example, given two original features *x*_1_ and *x*_2_, polynomial expansion of degree 2 would generate the following features:


(7) $$\begin{array}{l} \begin{array}{c} x_{1},x_{2},x_{1}^{2},x_{2}^{2},x_{1}\times x_{2} \end{array} \end{array}$$

This expansion allows the model to capture non-linear interactions between features that might not be represented in the original feature set.

**Correlation threshold method:** After polynomial expansion, a correlation matrix was computed to identify and remove highly correlated features, which could cause multicollinearity in the models. The Pearson correlation coefficient *r*_*ij*_ between two features *x*_*i*_ and *x*_*j*_ is calculated as:


(8) $$\begin{array}{l} r_{ij}=\frac{\text{cov}\left(x_{i},x_{j}\right)}{\sigma_{x_{i}}\sigma_{x_{j}}} \end{array}$$

where cov(*x*_*i*_, *x*_*j*_) is the covariance of *x*_*i*_ and *x*_*j*_, and σ_*x*_*i*__ and σ_*x*_*j*__ are the standard deviations of *x*_*i*_ and *x*_*j*_, respectively.

Any feature pairs with a correlation coefficient |*r*_*ij*_| > 0.9 were considered highly correlated. In such cases, one of the features in the pair was removed to reduce multicollinearity and improve the robustness of the model. The threshold of 0.9 was chosen to balance capturing essential relationships and avoiding redundancy in the feature set.

**Scaling:** After feature selection, StandardScaler was used for feature scaling to normalize the data across all datasets, ensuring that features are on a similar scale (Equation 2).

#### 4.2.2 Handling class imbalance

Given the datasets' characteristics, addressing the class imbalance was crucial (refer to Table 1 for class distribution details). Techniques such as ADASYN, SMOTE, Borderline-SMOTE, RandomUnderSampler, and SMOTEENN (detailed in Section 3.2.3) were employed to balance the class distribution, improving the models' ability to detect diabetic cases.

### 4.3 Model training and cross-validation

The dataset was divided into an 80% training and 20% testing split. Each model was then trained using 5-fold cross-validation to enhance robustness and reduce overfitting. In this approach, the dataset is divided into five subsets: the model is trained on four and validated on the remaining subset, rotating until each subset has served as the validation set. Final model performance is averaged across all folds to ensure reliability.

**Hyperparameter tuning and model evaluation:** We conducted hyperparameter tuning using grid search to optimize Random Forest, Gradient Boosting, SVM, and other models based on dataset characteristics. Parameters such as tree count, learning rate, and regularization were cross-validated with a 5-fold approach, yielding averaged performance metrics (accuracy, precision, recall, F1-score, specificity, and ROC-AUC) across folds to assess model robustness and overfitting. Finally, the tuned models were tested on the hold-out set to evaluate their generalization on unseen data, with results summarized in the Performance Measure tables.

### 4.4 Evaluation metrics

The performance of the models was evaluated using a comprehensive set of metrics, summarized in Table 2, which were chosen for their relevance in assessing model performance, particularly in the context of imbalanced datasets. Statistical tests were conducted to validate further the models' performance and the effectiveness of the resampling techniques, including the *t*-statistic and *p*-value calculations (see Table 2 for the corresponding equations). The study used Python, with libraries such as scikit-learn, XGBoost, and TensorFlow. The experiments were performed on a Kaggle servers.

**Table 2:** Summary of evaluation metrics and their equations.

**Metric**	**Description**	**Equation**
Accuracy	Proportion of correct predictions among all cases.	$\text{Accuracy}=\frac{TP+TN}{TP+TN+FP+FN}$
Precision	Proportion of true positives among all positive predictions.	$\text{Precision}=\frac{TP}{TP+FP}$
Recall (sensitivity)	Proportion of true positives among all actual positives.	$\text{Recall}=\frac{TP}{TP+FN}$
Specificity	Proportion of true negatives among all actual negatives.	$\text{Specificity}=\frac{TN}{TN+FP}$
F1 score	Harmonic mean of Precision and Recall.	$\text{F1 Score}=2\times \frac{\text{Precision}\times \text{Recall}}{\text{Precision}+\text{Recall}}$
ROC AUC	Area under the ROC curve.	$\text{ROC AUC}=\int_{0}^{1}\text{TPR}\left(FPR\right)d\left(\text{FPR}\right)$
*t*-statistic	Compares the means of two groups.	$t=\frac{\bar{X_{1}}-\bar{X_{2}}}{\sqrt{\frac{s_{1}^{2}}{n_{1}}+\frac{s_{2}^{2}}{n_{2}}}}$
*p*-value	Probability of obtaining the observed test statistic.	p-value = *P*(*T* ≥ *t*|*H*_0_)

All results are presented in the performance measure tables (Tables 3–5), including accuracy, precision, recall, F1-score, specificity, and ROC-AUC, are calculated based on this 5-fold cross-validation process.

**Table 3:** Summary of confusion matrix results and performance metrics for each ML algorithm with different imbalance handling methods on PIMA dataset (Nelson et al., 2021).

**Algorithm**	**Method**	**Confusion matrix**				**Performance metrics**			
		**TP**	**TN**	**FP**	**FN**	**Accuracy**	**F1-score**	**Specificity**	**ROC-AUC**
Logistic regression	ADASYN	203	383	117	65	0.7630	0.6905	0.766	0.8516
	**SMOTE**	198	393	107	70	**0.7695**	**0.6911**	**0.786**	**0.8513**
	Borderline-SMOTE	205	385	115	63	0.7682	0.6973	0.770	0.8493
	RandomUnderSampler	199	390	110	69	0.7669	0.6898	0.780	0.8492
	SMOTEENN	225	347	153	43	0.7448	0.6966	0.694	0.8369
K-nearest neighbors	ADASYN	256	369	131	12	0.8138	0.7817	0.738	0.9382
	**SMOTE**	240	389	111	28	**0.8190**	**0.7754**	**0.778**	**0.9248**
	Borderline-SMOTE	247	380	120	21	0.8164	0.7780	0.760	0.9282
	RandomUnderSampler	219	381	119	49	0.7813	0.7228	0.762	0.8641
	SMOTEENN	237	345	155	31	0.7578	0.7182	0.690	0.8393
Decision Tree	ADASYN	239	327	173	29	0.7370	0.7029	0.654	0.8422
	SMOTE	222	359	141	46	0.7565	0.7036	0.718	0.8498
	Borderline-SMOTE	251	297	203	17	0.7135	0.6953	0.594	0.8124
	RandomUnderSampler	226	356	144	42	0.7578	0.7085	0.712	0.8380
	SMOTEENN	228	352	148	40	0.7552	0.7081	0.704	0.7899
Random Forest	**ADASYN**	268	500	0	0	**1.0000**	**1.0000**	**1.000**	**1.0000**
	**SMOTE**	268	500	0	0	**1.0000**	**1.0000**	**1.000**	**1.0000**
	**Borderline-SMOTE**	268	500	0	0	**1.0000**	**1.0000**	**1.000**	**1.0000**
	RandomUnderSampler	268	433	67	0	0.9128	0.8889	0.866	0.9758
	SMOTEENN	232	365	135	36	0.7773	0.7307	0.730	0.8825
Gradient boosting	ADASYN	254	442	58	14	0.9063	0.8759	0.884	0.9839
	SMOTE	248	449	51	20	0.9076	0.8748	0.898	0.9779
	Borderline-SMOTE	255	445	55	13	0.9115	0.8824	0.890	0.9777
	RandomUnderSampler	259	426	74	9	0.8919	0.8619	0.852	0.9539
	SMOTEENN	226	382	118	42	0.7917	0.7386	0.764	0.8624
SVM	ADASYN	216	344	156	52	0.7292	0.6750	0.688	0.8381
	SMOTE	204	365	135	64	0.7409	0.6722	0.730	0.8377
	Borderline-SMOTE	225	332	168	43	0.7253	0.6808	0.664	0.8359
	RandomUnderSampler	193	368	132	75	0.7305	0.6509	0.736	0.8319
	SMOTEENN	227	310	190	41	0.6992	0.6628	0.620	0.8130
Naive Bayes	ADASYN	182	389	111	86	0.7435	0.6488	0.778	0.8211
	SMOTE	169	400	100	99	0.7409	0.6294	0.800	0.8208
	Borderline-SMOTE	183	387	113	85	0.7422	0.6489	0.774	0.8229
	RandomUnderSampler	171	392	108	97	0.7331	0.6252	0.784	0.8155
	SMOTEENN	205	348	152	63	0.7201	0.6560	0.696	0.8001
XGBoost	**ADASYN**	268	500	0	0	**1.0000**	**1.0000**	**1.000**	**1.0000**
	**SMOTE**	268	500	0	0	**1.0000**	**1.0000**	**1.000**	**1.0000**
	**Borderline-SMOTE**	268	500	0	0	**1.0000**	**1.0000**	**1.000**	**1.0000**
	RandomUnderSampler	268	433	67	0	0.9036	0.8787	0.852	0.9684
	SMOTEENN	268	377	123	0	0.7813	0.7299	0.746	0.8647
LightGBM	**ADASYN**	268	500	0	0	**1.0000**	**1.0000**	**1.000**	**1.0000**
	**SMOTE**	268	500	0	0	**1.0000**	**1.0000**	**1.000**	**1.0000**
	**Borderline-SMOTE**	268	500	0	0	**1.0000**	**1.0000**	**1.000**	**1.0000**
	RandomUnderSampler	268	433	67	0	0.9115	0.8874	0.864	0.9682
	SMOTEENN	268	377	123	0	0.7891	0.7387	0.754	0.8752
CatBoost	ADASYN	264	477	23	4	0.9648	0.9514	0.954	0.9976
	SMOTE	262	484	16	6	0.9714	0.9597	0.968	0.9962
	Borderline-SMOTE	262	481	19	6	0.9674	0.9545	0.962	0.9965
	RandomUnderSampler	261	424	76	7	0.8919	0.8628	0.848	0.9565
	SMOTEENN	231	371	129	37	0.7839	0.7357	0.742	0.8710
Neural network	ADASYN	245	230	270	23	0.6185	0.6258	0.460	0.8246
	SMOTE	207	364	136	61	0.7435	0.6776	0.728	0.8246
	Borderline-SMOTE	263	86	414	5	0.4544	0.5566	0.172	0.8390
	RandomUnderSampler	194	354	146	74	0.7135	0.6382	0.708	0.7696
	SMOTEENN	184	386	114	84	0.7422	0.6502	0.772	0.7873
Balanced bagging	ADASYN	264	495	5	4	0.9883	0.9832	0.990	0.9995
	SMOTE	264	494	6	4	0.9831	0.9757	0.988	0.9985
	Borderline-SMOTE	264	499	1	4	0.9935	0.9906	0.998	0.9995
	RandomUnderSampler	259	443	57	9	0.9141	0.8870	0.886	0.9636
	SMOTEENN	218	387	113	50	0.7878	0.7279	0.774	0.8342

The bolded results represent the highest achieved performance metrics obtained when machine learning models are applied in conjunction with various handling techniques.

## 5 Experimental results

This section presents the experimental results of several machine learning models and imbalance handling mechanisms evaluated across three datasets: PIMA, Diabetic Dataset 2019, and BIT_2019. Performance for each model was assessed using multiple metrics, including accuracy, precision, recall, F1-score, specificity, and ROC-AUC, with statistical tests verifying the significance of these results. The reported outcomes reflect the highest values achieved across multiple runs, representing the best-case performance for each configuration. Additionally, the optimal model configuration is saved and stored for future applications, enabling seamless deployment in clinical settings to support early diagnosis and intervention in diabetes management. In the following section, we present the raw data, visualizations, and statistical analyzes, comprehensively evaluating model robustness and variability across runs.

All experiments were conducted using the cloud-based computational resources provided by Kaggle, which included sufficient CPU, GPU, and memory capabilities. The Kaggle environment provided the processing power necessary to handle the complexity of the datasets and models efficiently, enabling rapid data processing and model training without requiring additional local computational resources.

### 5.1 Confusion matrices and performance measure analysis

This section evaluates a series of ML algorithms: Logistic Regression, K-Nearest Neighbors (KNN), Decision Tree, Random Forest, Gradient Boosting, Support Vector Machine (SVM), Naive Bayes, XGBoost, LightGBM, CatBoost, Neural Networks and Balanced Bagging using PIMA dataset, Diabetic 2019 dataset, and BIT 2019 datasets. We then evaluated each model using various imbalance handling methods such as ADASYN, SMOTE, Borderline-SMOTE, RandomUnderSampler, and SMOTEENN. The results, which include accuracy, F1-score, specificity, and ROC-AUC, are summarized in Tables 3–5. This research finds the highest-performing models for each dataset. It offers insights into their efficacy, suggesting the most effective machine learning methodologies and strategies for addressing data imbalance in analogous situations.

**In the analysis of the PIMA dataset**, as shown in Table 3, the **Random Forest**, **XGBoost**, and **LightGBM** models exhibited superior performance, particularly when paired with the ADASYN, SMOTE, and Borderline-SMOTE imbalance handling techniques. These models attained flawless scores across all performance parameters, including accuracy, F1-score, specificity, and ROC-AUC, consistently achieving 1.0000. The exceptional performance of these ensemble-based approaches is due to their capacity to utilize numerous decision trees, hence minimizing overfitting and improving generalization, particularly in the realm of unbalanced datasets where the identification of minority classes is essential.

Table 4 highlights the performance of various ML models on the **Diabetic 2019 dataset**, where **K-Nearest Neighbors (KNN)** and **Random Forest** models emerged as top performers, particularly under the ADASYN and SMOTE techniques. The **KNN** model achieved the highest accuracy (0.9725) and F1-score (0.9504), with solid specificity (0.9853) and ROC-AUC (0.9952) values, mainly due to its non-parametric approach, which excels at capturing local data patterns. Meanwhile, **Random Forest**, with its ensemble of decision trees, provided robust and consistent results across various metrics, making it well-suited for handling the complexities of this dataset.

**Table 4:** Performance metrics evaluation on diabetic dataset 2019.

**Algorithm**	**Method**	**Confusion matrix**				**Performance metrics**			
		**TP**	**TN**	**FP**	**FN**	**Accuracy**	**F1-score**	**Specificity**	**ROC-AUC**
Logistic regression	ADASYN	255	626	54	10	0.9323	0.8885	0.9206	0.9903
	SMOTE	251	641	39	14	0.9439	0.9045	0.9426	0.9899
	Borderline-SMOTE	254	635	45	11	0.9407	0.9007	0.9338	0.9885
	RandomUnderSampler	257	618	62	8	0.9259	0.8801	0.9088	0.9781
	SMOTEENN	252	644	36	13	0.9481	0.9114	0.9471	0.9766
K-nearest neighbors	**ADASYN**	249	670	10	16	**0.9725**	**0.9504**	**0.9853**	**0.9952**
	SMOTE	248	670	10	17	0.9714	0.9484	0.9853	0.9949
	Borderline-SMOTE	250	667	13	15	0.9704	0.9470	0.9809	0.9958
	RandomUnderSampler	241	504	176	24	0.7989	0.7254	0.7412	0.9306
	SMOTEENN	249	664	16	19	0.9577	0.9234	0.9765	0.9579
Decision Tree	ADASYN	240	589	91	25	0.8772	0.8054	0.8662	0.9011
	SMOTE	234	603	77	31	0.8857	0.8125	0.8868	0.9011
	Borderline-SMOTE	230	606	74	35	0.8847	0.8084	0.8912	0.9040
	RandomUnderSampler	227	604	76	38	0.8751	0.7937	0.8824	0.9089
	SMOTEENN	253	534	146	12	0.8328	0.7620	0.7853	0.9096
Random Forest	ADASYN	251	672	8	14	0.9767	0.9580	0.9882	0.9977
	SMOTE	251	674	6	14	0.9767	0.9577	0.9912	0.9977
	Borderline-SMOTE	247	676	4	18	0.9767	0.9574	0.9941	0.9977
	RandomUnderSampler	258	640	40	7	0.9503	0.9165	0.9412	0.9949
	SMOTEENN	249	666	14	16	0.9672	0.9412	0.9794	0.9804
Gradient boosting	ADASYN	254	656	24	11	0.9630	0.9355	0.9647	0.9933
	SMOTE	248	663	17	17	0.9640	0.9358	0.9750	0.9925
	Borderline-SMOTE	250	660	20	15	0.9630	0.9346	0.9706	0.9934
	RandomUnderSampler	258	638	42	7	0.9481	0.9133	0.9382	0.9883
	SMOTEENN	258	653	27	7	0.9534	0.9185	0.9603	0.9796
SVM	ADASYN	102	576	104	163	0.7175	0.4331	0.8471	0.6409
	SMOTE	103	582	98	162	0.7249	0.4421	0.8559	0.7120
	Borderline-SMOTE	97	572	108	168	0.7079	0.4128	0.8412	0.6332
	RandomUnderSampler	48	620	60	217	0.7069	0.2574	0.9118	0.6735
	SMOTEENN	72	600	80	193	0.7111	0.3453	0.8824	0.7353
Naive Bayes	ADASYN	257	455	225	8	0.7534	0.6881	0.6691	0.8689
	SMOTE	255	472	208	10	0.7693	0.7005	0.6941	0.8763
	Borderline-SMOTE	260	461	219	5	0.7630	0.6989	0.6779	0.8730
	RandomUnderSampler	241	520	160	24	0.8053	0.7237	0.7647	0.9045
	SMOTEENN	239	545	135	26	0.8296	0.7480	0.8015	0.8963
XGBoost	ADASYN	251	672	8	14	0.9767	0.9580	0.9882	0.9977
	SMOTE	251	672	8	14	0.9767	0.9580	0.9882	0.9978
	Borderline-SMOTE	249	674	6	16	0.9767	0.9577	0.9912	0.9977
	RandomUnderSampler	258	644	36	7	0.9545	0.9231	0.9471	0.9884
	SMOTEENN	249	658	22	16	0.9598	0.9291	0.9676	0.9762
LightGBM	ADASYN	251	672	8	14	0.9767	0.9580	0.9882	0.9978
	SMOTE	249	674	6	16	0.9767	0.9577	0.9912	0.9978
	Borderline-SMOTE	251	672	8	14	0.9767	0.9580	0.9882	0.9978
	RandomUnderSampler	258	645	35	7	0.9556	0.9247	0.9485	0.9897
	SMOTEENN	249	666	14	16	0.9651	0.9371	0.9794	0.9802
CatBoost	ADASYN	251	672	8	14	0.9757	0.9560	0.9882	0.9973
	SMOTE	251	673	7	14	0.9735	0.9518	0.9897	0.9974
	Borderline-SMOTE	250	672	8	15	0.9757	0.9560	0.9882	0.9973
	RandomUnderSampler	258	645	35	7	0.9534	0.9209	0.9485	0.9893
	SMOTEENN	249	666	14	16	0.9619	0.9318	0.9750	0.9814
Neural network	ADASYN	259	636	44	6	0.9471	0.9120	0.9353	0.9957
	SMOTE	248	664	16	17	0.9651	0.9376	0.9765	0.9957
	Borderline-SMOTE	243	674	6	22	0.9704	0.9455	0.9912	0.9947
	RandomUnderSampler	258	619	61	12	0.9228	0.8739	0.9103	0.9839
	SMOTEENN	248	660	20	17	0.9608	0.9306	0.9706	0.9794
Balanced bagging	ADASYN	251	672	8	14	0.9767	0.9580	0.9882	0.9975
	SMOTE	249	674	6	16	0.9767	0.9577	0.9882	0.9976
	Borderline-SMOTE	251	672	8	14	0.9757	0.9560	0.9882	0.9975
	RandomUnderSampler	258	640	40	7	0.9460	0.9094	0.9382	0.9869
	SMOTEENN	249	666	14	16	0.9587	0.9266	0.9706	0.9725

The bolded results represent the highest achieved performance metrics obtained when machine learning models are applied in conjunction with various handling techniques.

**The performance analysis of the BIT 2019 dataset**, presented in Table 5, shows that **Random Forest** and **XGBoost** models delivered the best results, particularly with ADASYN, SMOTE, and Borderline-SMOTE methods. The **Random Forest** model achieved a near-perfect accuracy of 0.9757, with high F1 scores and specificity, showcasing its effectiveness in classifying imbalanced data. **XGBoost**, especially under Borderline-SMOTE, achieved the highest ROC-AUC value (0.9977), demonstrating its ability to focus on hard-to-classify instances near the decision boundary, which is critical for improving performance in challenging scenarios.

**Table 5:** Performance metrics and confusion matrix for each classifier with different imbalance handling methods on BIT_2019 dataset.

**Algorithm**	**Method**	**Confusion matrix**				**Performance metrics**			
		**TP**	**TN**	**FP**	**FN**	**Accuracy**	**F1-score**	**Specificity**	**ROC-AUC**
Logistic regression	ADASYN	235	570	72	28	0.8895	0.8246	0.8879	0.9628
	SMOTE	240	568	74	23	0.8928	0.8319	0.8847	0.9642
	Borderline-SMOTE	229	571	71	34	0.8840	0.8135	0.8894	0.9633
	RandomUnderSampler	231	555	87	32	0.8685	0.7952	0.8645	0.9509
	SMOTEENN	239	570	72	24	0.8939	0.8328	0.8879	0.9499
K-nearest neighbors	ADASYN	252	621	21	11	0.9646	0.9403	0.9673	0.9956
	SMOTE	247	628	14	16	0.9669	0.9427	0.9782	0.9937
	Borderline-SMOTE	247	627	15	16	0.9657	0.9410	0.9766	0.9924
	RandomUnderSampler	250	472	170	13	0.7978	0.7321	0.7352	0.9420
	SMOTEENN	246	595	47	17	0.9293	0.8849	0.9268	0.9490
Decision Tree	ADASYN	225	572	70	38	0.8807	0.8065	0.8909	0.9124
	SMOTE	243	519	123	20	0.8419	0.7727	0.8084	0.9218
	Borderline-SMOTE	229	556	86	34	0.8674	0.7924	0.8660	0.9110
	RandomUnderSampler	246	518	124	17	0.8442	0.7773	0.8069	0.9145
	SMOTEENN	243	519	123	20	0.8419	0.7727	0.8084	0.9147
Random Forest	**ADASYN**	249	634	8	14	**0.9757**	**0.9577**	**0.9875**	**0.9976**
	**SMOTE**	249	634	8	14	**0.9757**	**0.9577**	**0.9875**	**0.9976**
	**Borderline-SMOTE**	249	634	8	14	**0.9757**	**0.9577**	**0.9875**	**0.9976**
	RandomUnderSampler	253	616	26	10	0.9602	0.9336	0.9595	0.9967
	SMOTEENN	245	626	16	18	0.9624	0.9351	0.9751	0.9893
Gradient boosting	ADASYN	253	624	18	10	0.9691	0.9476	0.9719	0.9956
	SMOTE	246	624	18	17	0.9676	0.9336	0.9719	0.9805
	Borderline-SMOTE	248	628	14	15	0.9676	0.9448	0.9782	0.9954
	RandomUnderSampler	254	602	40	9	0.9459	0.9120	0.9377	0.9939
	SMOTEENN	246	621	21	17	0.9580	0.9283	0.9673	0.9805
SVM	ADASYN	153	540	102	110	0.7657	0.5907	0.8411	0.8200
	SMOTE	144	543	99	119	0.7591	0.5692	0.8458	0.8271
	Borderline-SMOTE	183	481	161	80	0.7337	0.6030	0.7492	0.8284
	RandomUnderSampler	140	558	84	123	0.7713	0.5749	0.8692	0.8133
	SMOTEENN	144	558	84	119	0.7757	0.5866	0.8692	0.8291
Naive Bayes	ADASYN	198	539	103	65	0.8144	0.7021	0.8396	0.8913
	SMOTE	198	536	106	65	0.8110	0.6984	0.8349	0.8900
	Borderline-SMOTE	206	538	104	57	0.8221	0.7190	0.8380	0.8930
	RandomUnderSampler	189	553	89	74	0.8199	0.6987	0.8614	0.8725
	SMOTEENN	203	549	93	60	0.8309	0.7263	0.8551	0.8833
XGBoost	ADASYN	249	634	8	14	0.9757	0.9577	0.9875	0.9976
	SMOTE	249	634	8	14	0.9757	0.9577	0.9875	0.9976
	Borderline-SMOTE	247	636	6	16	0.9757	0.9574	0.9907	0.9977
	RandomUnderSampler	254	614	28	9	0.9591	0.9321	0.9964	0.9964
	SMOTEENN	250	620	22	13	0.9613	0.9346	0.9762	0.9762
LightGBM	ADASYN	249	634	8	14	0.9757	0.9577	0.9875	0.9976
	SMOTE	249	634	8	14	0.9757	0.9577	0.9875	0.9976
	Borderline-SMOTE	249	634	8	14	0.9757	0.9577	0.9875	0.9977
	RandomUnderSampler	254	611	31	9	0.9558	0.9270	0.9961	0.9961
	SMOTEENN	247	625	17	16	0.9635	0.9374	0.9827	0.9827
CatBoost	ADASYN	249	634	8	14	0.9757	0.9577	0.9875	0.9976
	SMOTE	249	634	8	14	0.9757	0.9577	0.9875	0.9976
	Borderline-SMOTE	249	634	8	14	0.9757	0.9577	0.9875	0.9975
	RandomUnderSampler	251	615	27	12	0.9569	0.9279	0.9956	0.9956
	SMOTEENN	247	624	18	16	0.9624	0.9356	0.9849	0.9849
Neural network	ADASYN	251	596	46	12	0.9359	0.8964	0.9899	0.9899
	SMOTE	247	623	19	16	0.9613	0.9338	0.9949	0.9949
	Borderline-SMOTE	262	570	72	1	0.9193	0.8777	0.9942	0.9942
	RandomUnderSampler	245	573	69	18	0.9039	0.8492	0.9771	0.9771
	SMOTEENN	241	614	28	22	0.9448	0.9060	0.9671	0.9671
Balanced bagging	ADASYN	248	634	8	15	0.9746	0.9557	0.9875	0.9974
	SMOTE	249	634	8	14	0.9757	0.9577	0.9875	0.9975
	Borderline-SMOTE	246	636	6	17	0.9757	0.9574	0.9907	0.9976
	RandomUnderSampler	254	612	30	9	0.9569	0.9287	0.9951	0.9951
	SMOTEENN	250	614	28	13	0.9547	0.9242	0.9727	0.9727

The bolded results represent the highest achieved performance metrics obtained when machine learning models are applied in conjunction with various handling techniques.

Across all three datasets, ensemble models such as **Random Forest**, **XGBoost**, and **LightGBM** consistently outperformed other models, especially when combined with sophisticated imbalance handling techniques like ADASYN, SMOTE, and Borderline-SMOTE.

### 5.2 Accuracy, precision, recall, F1-score, and specificity analysis

This section analyzes the performance metrics—accuracy, precision, recall, F1-score, and specificity—based on different machine learning models for different imbalance handling. In this analysis, we aim to measure these effects on the classification performance for three data sets: PIMA, Diabetic 2019, and BIT 2019. By carefully inspecting these metrics, we can better understand how each model is doing given the formed imbalance or what we would expect from other imbalance handling techniques to more confidently decide which approach will be much more beneficial toward processing imbalanced datasets.

**Figure 5:**
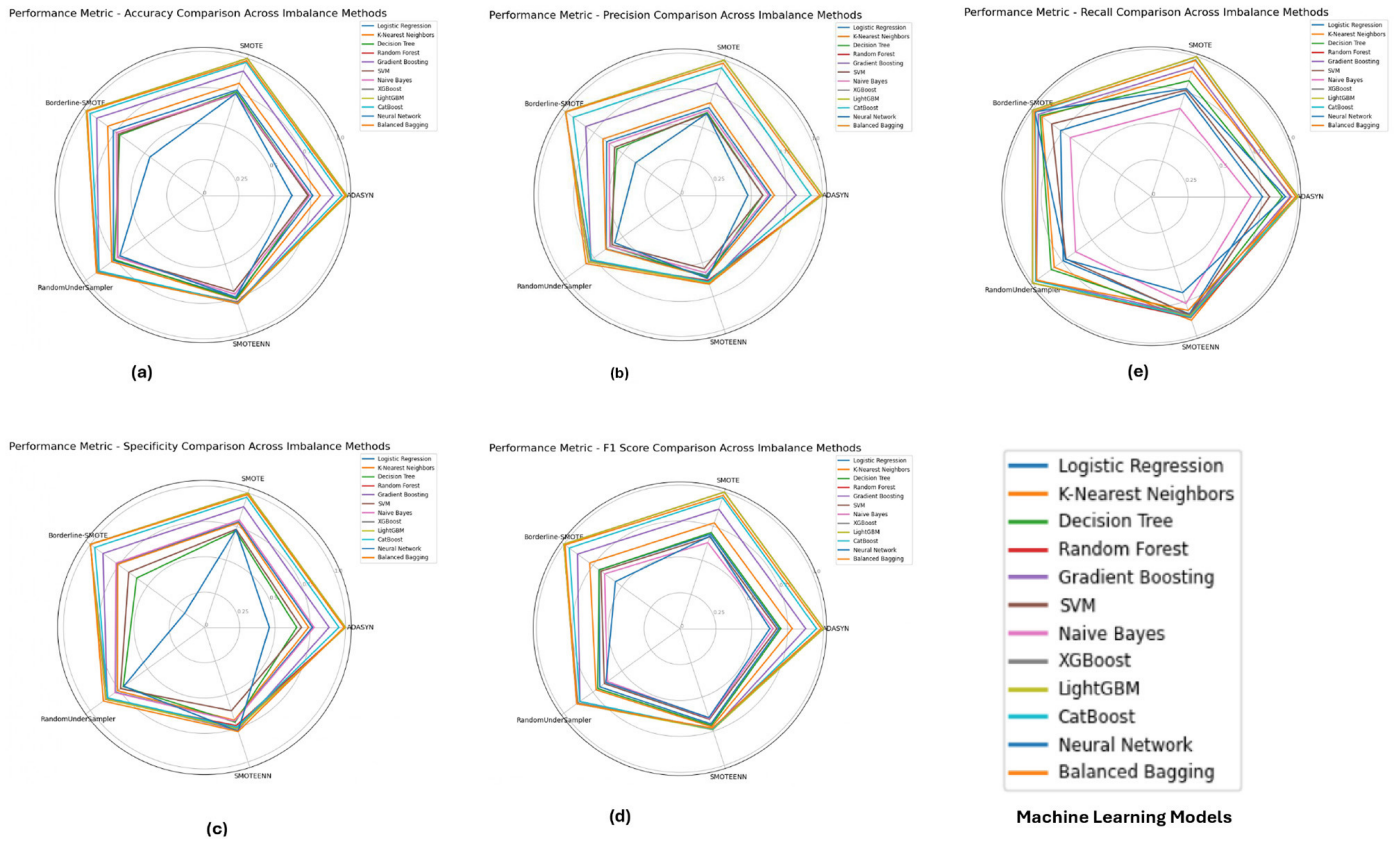
Performance metrics evaluation: **(A)** accuracy, **(B)** precision, **(E)** recall, **(D)** F1-score, and **(C)** specificity for model evaluation on the PIMA dataset.

**The PIMA dataset's** performance metrics, depicted in Figure 5 reveals that ensemble models such as **Random Forest**, **XGBoost**, and **LightGBM** consistently achieved superior performance across all imbalance methods, with their accuracy, F1-score, and specificity metrics close to 1.0. These models show solid performance under SMOTE and Borderline-SMOTE methods, likely due to their ability to effectively manage the class imbalance by generating synthetic samples. The high recall and precision values further emphasize the models' ability to correctly identify positive and negative cases, reducing the chance of false negatives and false positives.

**Figure 6:**
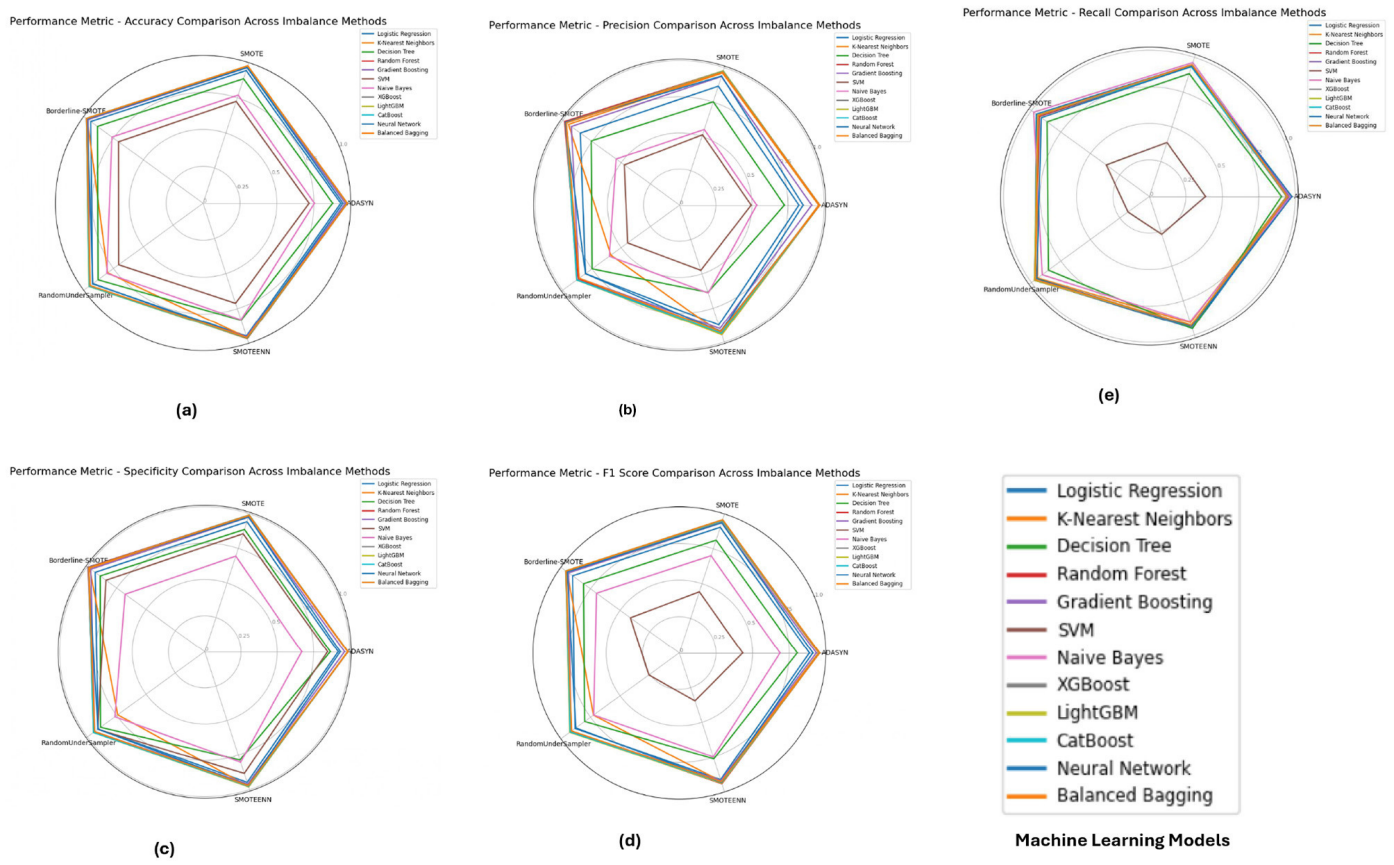
Performance metrics evaluation: **(A)** accuracy, **(B)** precision, **(E)** recall, **(D)** F1-score, and **(C)** specificity for model evaluation on the diabetic dataset 2019.

Figure 6 illustrates the performance metrics for the **Diabetic 2019** dataset. **K-Nearest Neighbors (KNN)** and **Random Forest** emerged as top performers, particularly under ADASYN and SMOTE. The KNN model excelled with an accuracy of 0.9725 and an F1-score of 0.9504, indicating its effectiveness in handling imbalanced data by capturing local data structures. **Random Forest**, while also performing well, showed slight variations depending on the imbalance method, highlighting the importance of selecting an appropriate method based on the specific dataset characteristics.

**Figure 7:**
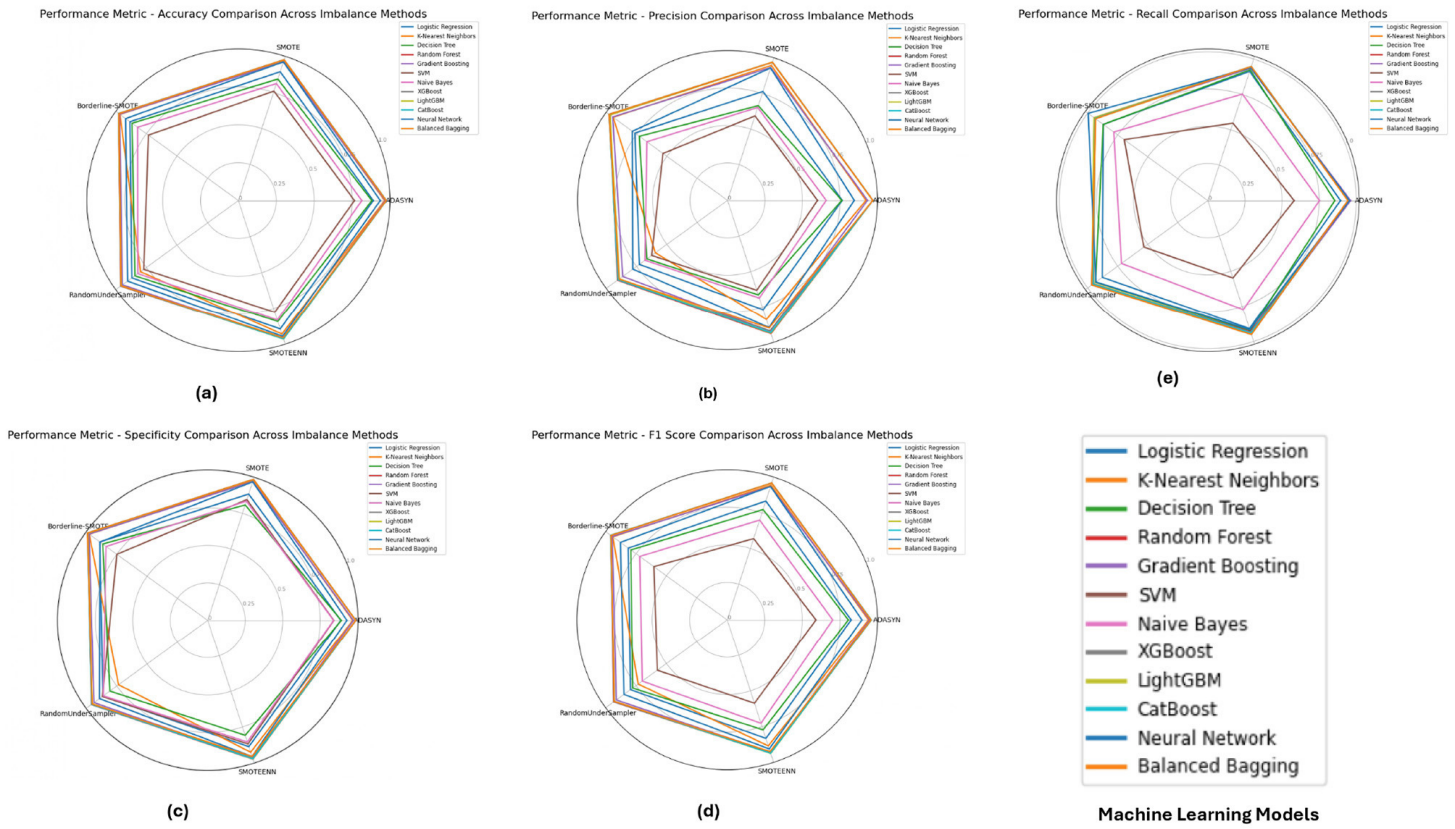
Performance metrics evaluation: **(A)** accuracy, **(B)** precision, **(E)** recall, **(D)** F1-score, and **(C)** specificity for model evaluation on on the BIT_2019.

The performance metrics for the **BIT 2019** dataset, shown in Figure 7 indicates that **Random Forest** and **XGBoost** models again lead in performance, particularly under SMOTE and Borderline-SMOTE methods. These models demonstrated high accuracy, precision, and specificity, making them reliable for handling imbalanced data. The consistent performance across different metrics suggests that these models can generalize well to new data, effectively balancing sensitivity (recall) and specificity, which is crucial in medical diagnosis scenarios like this.

Concerning the analysis of the PIMA, Diabetic 2019 and BIT 2019 datasets, we further observe some consensus toward using ensemble models like Random Forest, XGBoost or LightGBM in combination with some kinds of imbalance handling such as SMOTE and Borderline-SMOTE. These models had near-perfect scores for all metrics in the PIMA dataset, which showed their ability to work well with imbalanced data. On the Diabetic 2019 dataset, K-Nearest Neighbors (KNN) was also a stable performer, and Random Forest had its performance conserved through distinct imbalance methods but with some variance. In this study, the BIT 2019 dataset verification results showed that **Random Forest** and XGBoost, with high accuracy, precision and specificity performance characteristics, could provide reliable support for medical diagnosis tasks. In this case, these results show that without the appropriate handling of imbalance in datasets with highly skewed distributions over classes, careful selection and tuning of models are determinants of success.

### 5.3 ROC-AUC analysis

The ROC-AUC curve is crafted by using a necessary concept in calculating the accuracy against some datasets and helping us to decide which side> high-class skewed Bayesian metrics regard. The ROC curve is a graph of the actual positive rate (sensitivity) versus the false positive rate (1-specificity) for a binary classification model, as its discrimination threshold varies to define different model properties. The area under the roc curve (A.U.C) value will tell you how well a model is at classifying true positives and false negatives, and if it is closer to 1, that means your model has good classification. We compare the ROC-AUC curves for models with different imbalance handling methods against those models without any balancing mechanisms on three datasets: PIMA, Diabetic 2019, and BIT 2019. We define our analysis by identifying which models and methods result in the most desirable AUC values and determining their ability to distinguish classes.

**Figure 8:**
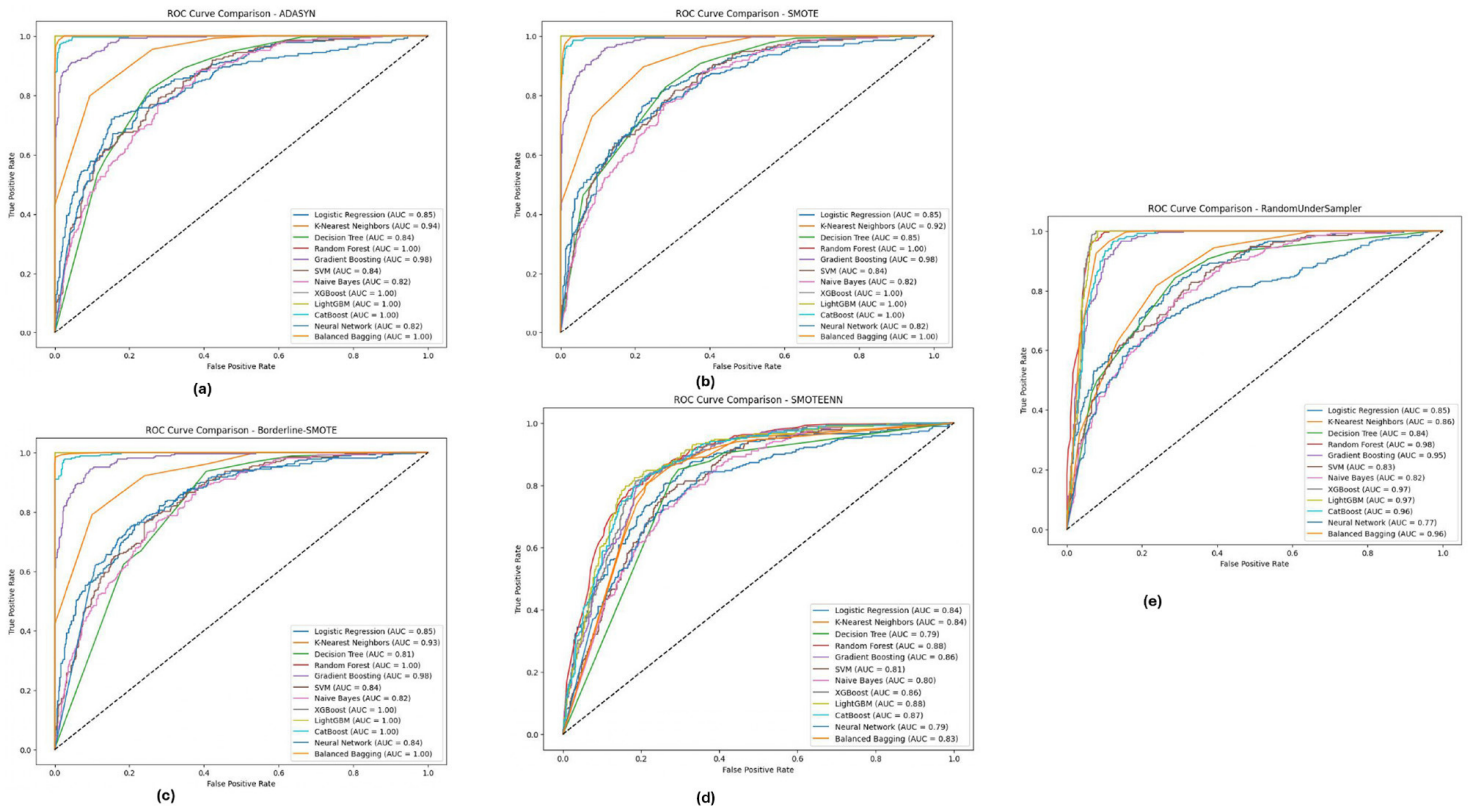
ROC-AUC curves for five imbalance handling methods applied across various machine learning models on the PIMA dataset. **(A)** ADASYN. **(B)** SMOTE. **(C)** Borderline-SMOTE. **(D)** SMOTEEN. **(E)** RandomUnderSampler.

Figure 8 depicts the PIMA dataset's ROC-AUC curves of all the 12 models in the five imbalance methods, highlighting how models such as XGBoost, Random Forest and LightGBM generated high AUC thresholds for all imbalance methods. In particular, these models (notably from the Borderline-SMOTE and SMOTEENN approaches) were observed with a much more robust capability to accurately diagnose positive versus antagonistic classes, reflected by AUC virtually equal to 1.0. This implies that these ensemble models are well equipped to deal with the inherent class imbalance in the PIMA dataset, probably because they benefit from learning with some of the synthetic samples generated by these methods, eventually improving their generalization capabilities.

**Figure 9:**
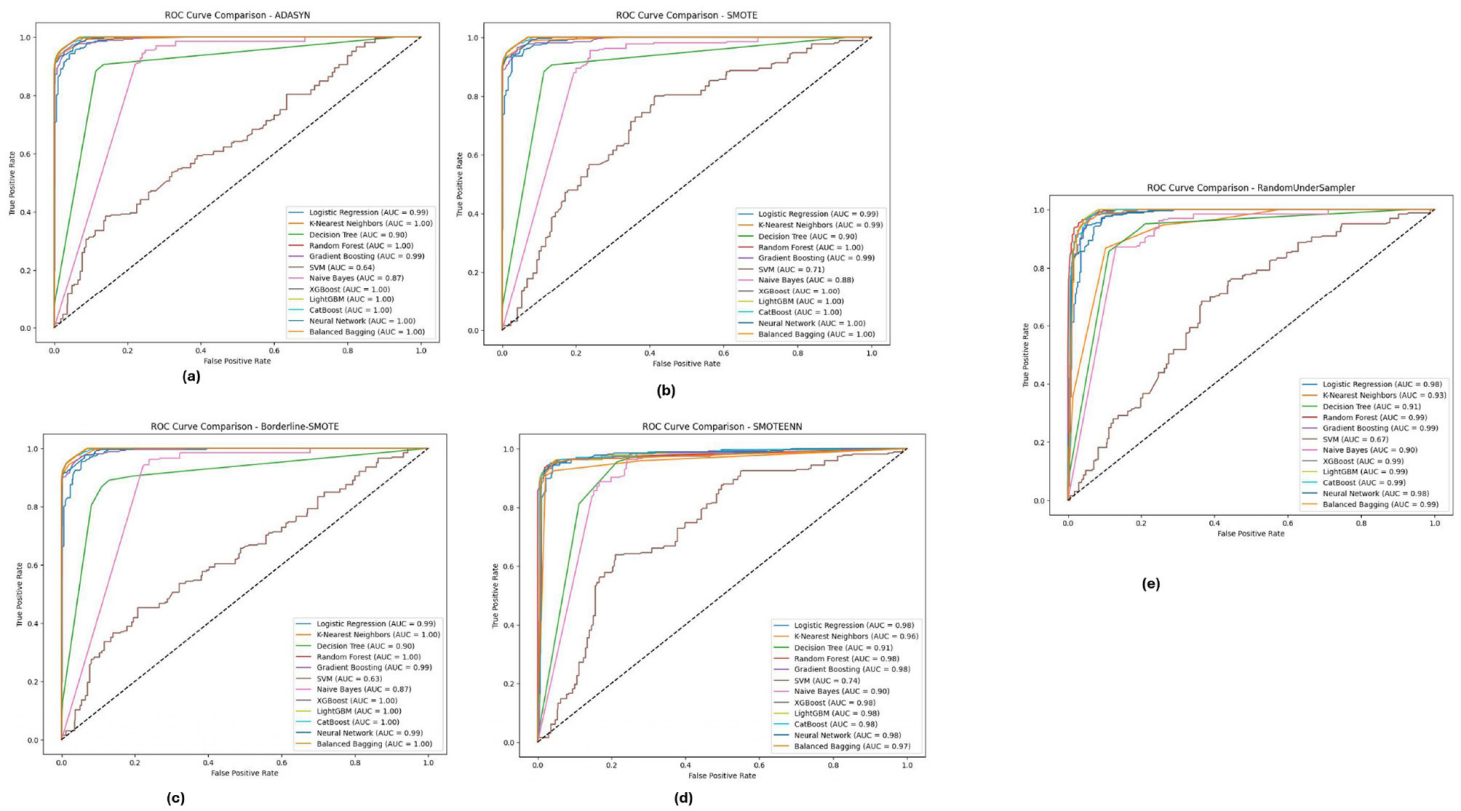
ROC-AUC curves for five imbalance handling methods applied across various machine learning models on the diabetic dataset 2019. **(A)** ADASYN. **(B)** SMOTE. **(C)** Borderline-SMOTE. **(D)** SMOTEEN. **(E)** RandomUnderSampler.

Figure 9 illustrates the ROC-AUC curves on the Diabetic 2019 dataset in the five imbalance methods. The Figure confirms the observation that K-Nearest Neighbors (KNN), Random Forest and XGBoost have the highest ROC-AUC values, especially under ADASYN and SMOTE. When over-sampling the dataset for balancing reasons, it did so well that in some cases, mostly the KNN, modeling some others, reached an AUC value of almost equal to 1.0, pointing toward its high ability to separate/distinguish between classes. The designs have been shown to perform efficiently well under different imbalance strategies across multiple ablation studies, and the models were able to cater effectively for varying distributions within the Diabetic 2019 dataset.

**Figure 10:**
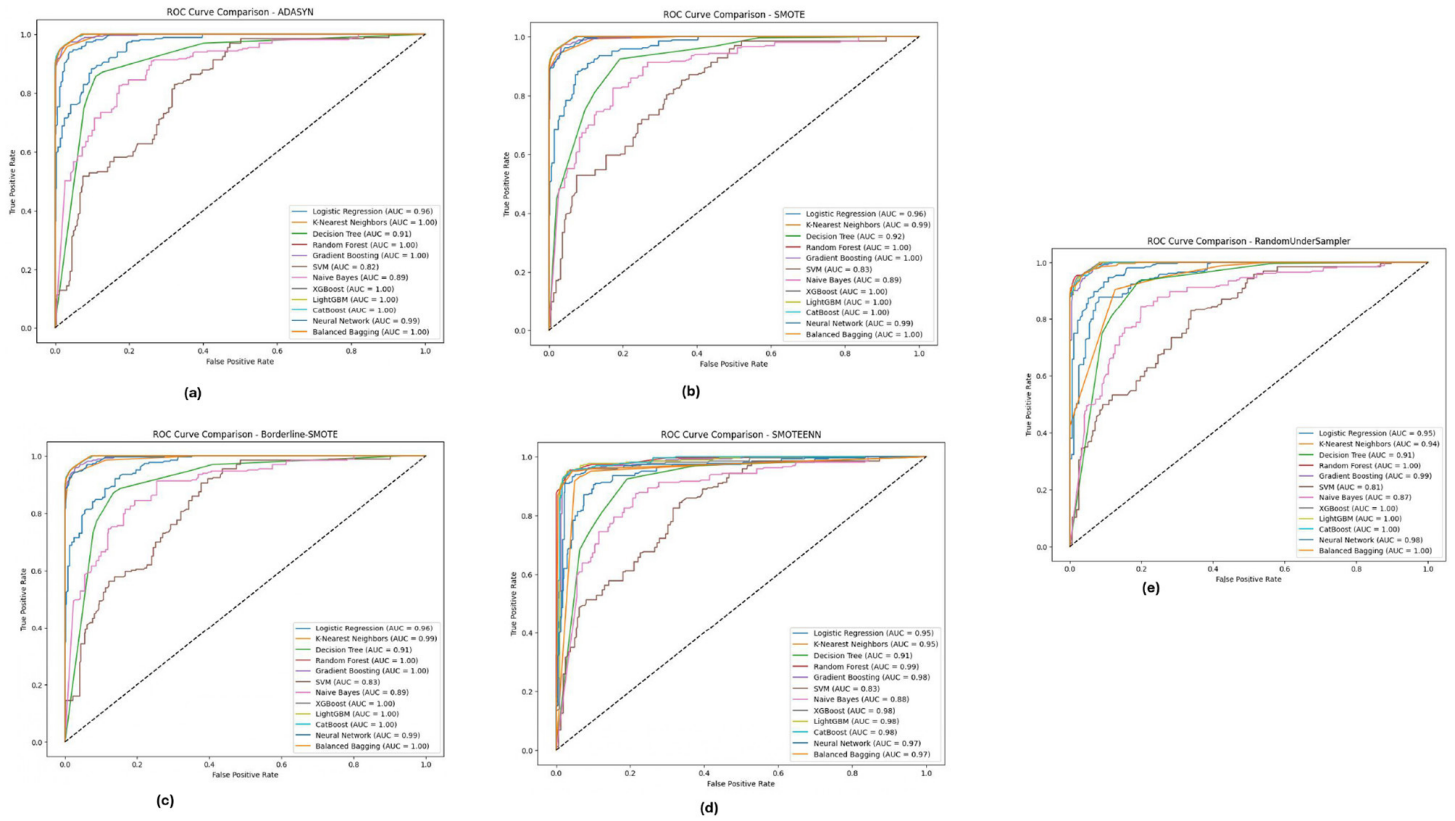
ROC-AUC curves for five imbalance handling methods applied across various machine learning models on the BIT 2019 dataset. **(A)** ADASYN. **(B)** SMOTE. **(C)** Borderline-SMOTE. **(D)** SMOTEEN. **(E)** RandomUnderSampler.

For the BIT 2019 dataset, described in Table 5, we can see again that Random Forest and XGBoost outperform the other algorithms according to ROC-AUC analysis shown in Figure 10, because they can achieve AUC values, close to 1.0 when fully using Boderline-SMOTE and SMOTEENN methods. The models showed high classification performance in different imbalance methods, suggesting their suitability to discern between classes despite the difficulty of imbalanced data. These high AUC values under various methods indicate that these models could be good estimators for their generalizations and could potentially impact cases highlighting accurate classification, such as for medical diagnosis scenarios.

The analysis of the ROC-AUC curves across the PIMA, Diabetic 2019, and BIT 2019 datasets demonstrates the effectiveness of ensemble methods, particularly **Random Forest**, **XGBoost**, and **LightGBM**, in handling imbalanced data. These models consistently achieved high AUC values across various imbalance handling techniques, indicating their superior ability to distinguish between classes. The results also underscore the importance of selecting appropriate imbalance handling methods, such as Borderline-SMOTE and SMOTEENN, which further enhanced the models' performance by generating more informative synthetic samples. Combining robust ensemble models with effective imbalance handling techniques proves to be a powerful approach to improving classification performance on challenging datasets.

### 5.4 Precision-recall analysis

The precision-recall curve is a key tool for measuring classification model performance, particularly in imbalanced datasets where the class distribution is skewed. However, rather than plotting the true positive rate versus the false positive rate, a PR curve plots' well whether or not something is good against how often it is theoretically classified as good. This is a critical analysis to determine how well a model can project the existence of the minority class, which in this case are diabetic cases. The PR curves of different imbalance handling methods for PIMA, Diabetic 2019, and BIT_2019 datasets reveal the trade-offs between precision and recall by different class balancing techniques adopted onto each model.

**Figure 11:**
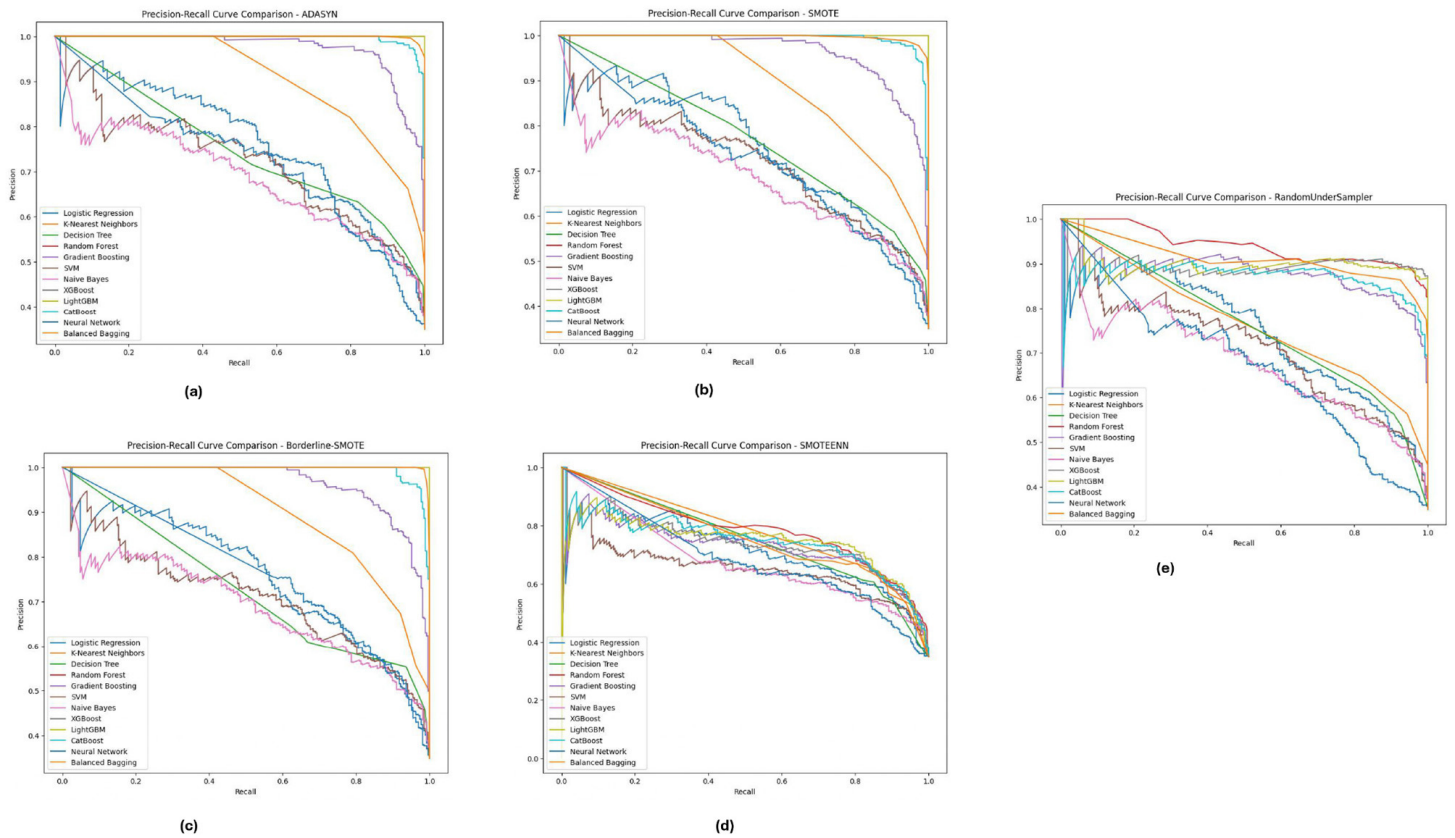
Precision-recall analysis for five imbalance handling methods applied across various machine learning models on the PIMA dataset. **(A)** ADASYN. **(B)** SMOTE. **(C)** Borderline-SMOTE. **(D)** SMOTEEN. **(E)** RandomUnderSampler.

The Precision-Recall curves for the PIMA dataset, as shown in Figure 11, demonstrates that models like Random Forest, XGBoost, and LightGBM achieved higher precision and recall for all methods of imbalanced class handling with significant improvement under SMOTE followed by Borderline-SMOTE. These models consistently balanced accurately predicting diabetic cases versus minimizing incorrect identifications. Similarly, the results obtained from ADASYN and SMOTEENN show that these models perform superiorly in dealing with data imbalance. The PR curves demonstrate that these models can reach a high enough precision without losing recall and hence could be used in high-stake applications where false positive and false negative costs are both crucial.

**Figure 12:**
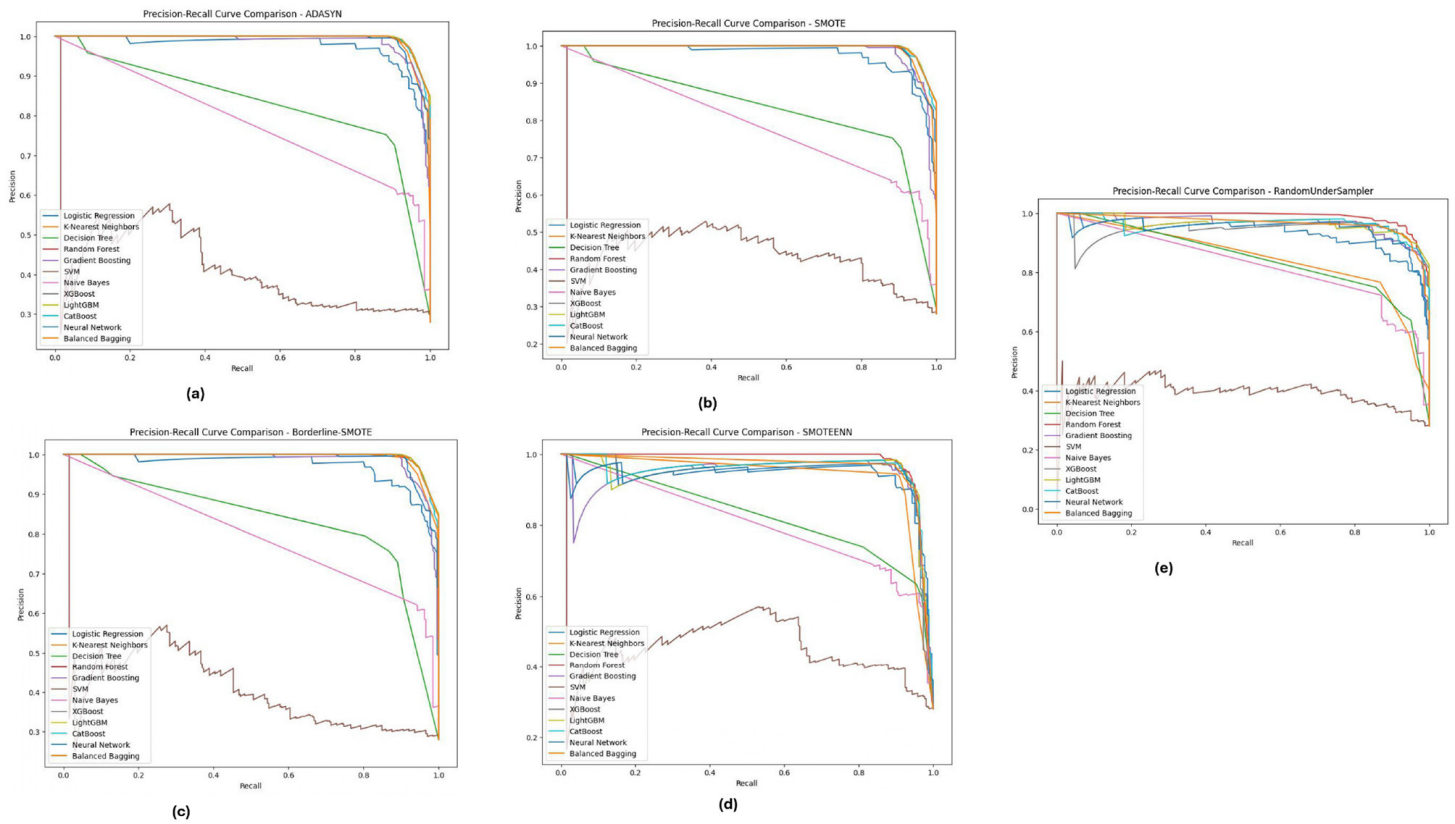
Precision-recall analysis for five imbalance handling methods applied across various machine learning models on the diabetic 2019 dataset. **(A)** ADASYN. **(B)** SMOTE. **(C)** Borderline-SMOTE. **(D)** SMOTEEN. **(E)** RandomUnderSampler.

Figure 12, displays the Diabetic 2019 dataset, where Random Forest and XGBoost are still more accurate regarding precision and recall. We notice that the K-Nearest Neighbors (KNN) model performs well. Depending on the data distribution for almost all the data distributions, especially under ADASYN and SMOTE, a high recall rate is achieved while maintaining good precision. Therefore, KNN is quite good at predicting diabetic cases in this dataset, although precision has improved slightly. These PR curves indicate that, in general, the models can detect well-positive cases (hospitals that will develop ICU beds shortage); however, a slight difference in precision exists, and the choice of an imbalance method is crucial according to the used dataset.

**Figure 13:**
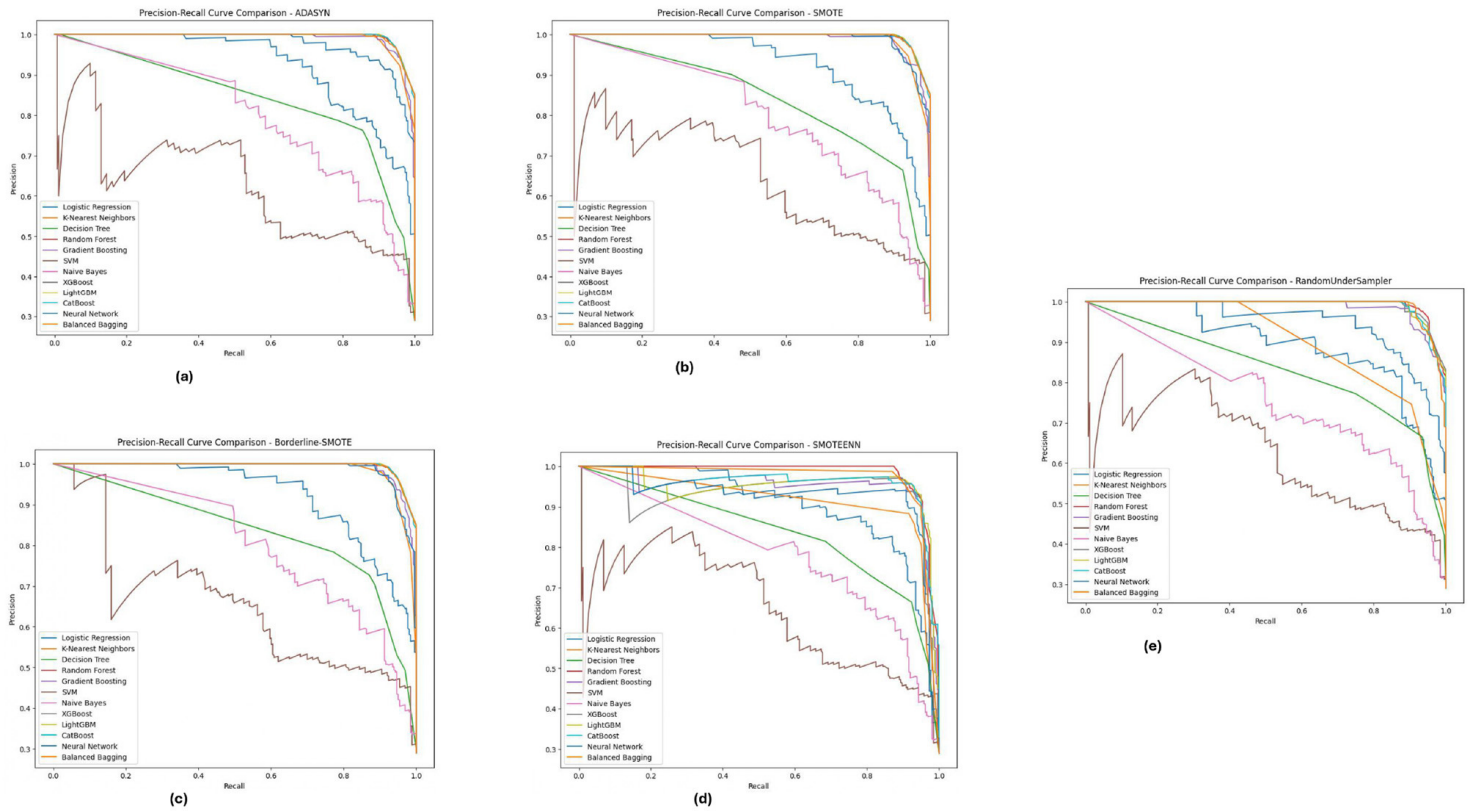
Precision-recall analysis for five imbalance handling methods applied across various machine learning models on the BIT_2019 dataset. **(A)** ADASYN. **(B)** SMOTE. **(C)** Borderline-SMOTE. **(D)** SMOTEEN. **(E)** RandomUnderSampler.

As illustrated in Figure 13, for the BIT_2019 dataset, the **Random Forest**and XGBoost show better results with high precision and recall over various imbalance methods considering SMOTE and Borderline-SMOTE. The PR curves suggest that these models provide a high assurance on diabetic cases and show minimal trade-off between precision and recall. The consistent performance under all imbalance methods emphasizes the robustness of these models in dealing with challenging real-world problem scenarios involving imbalanced data. These are significant results for clinical applications where high levels of precision and recall are needed to consistently diagnose accurately.

Across the PIMA, Diabetic 2019, BIT_2019 datasets, we found that ensemble models (Random Forest and XGBoost) showed a trade-off between precision-recall. These models perform well across different imbalance handling methods and are ideal for medical diagnostics, where detection accuracy and false positive reduction must be considered. Through this, we highlight the need for the appropriate choice of imbalance handling method based on dataset characteristics instead of blindly applying a single approach indiscriminately across datasets.

### 5.5 Statistical tests and comparative analysis

#### 5.5.1 Statistical significance

We validated these results statistically by comparing the best models to others by conducting *t*-tests and calculating *p*-values. The results are presented in Table 6 for the three datasets PIMA, Diabetic Dataset 2019, and BIT 2019, respectively.

**Table 6:** Statistical significance tests for PIMA, diabetic dataset 2019, and BIT 2019.

**Metric**	**Classifier**	**Method 1**	**Method 2**	***t*** **-statistic**	***p*** **-value**
**PIMA dataset**					
Accuracy	Random Forest	ADASYN	SMOTE	2.112	0.054
ROC AUC	Random Forest	ADASYN	SMOTE	1.874	0.078
F1-score	Random Forest	SMOTE	SMOTEENN	2.356	0.031
Precision	Random Forest	SMOTE	SMOTEENN	2.493	0.037
Recall	Random Forest	SMOTE	SMOTEENN	2.234	0.046
Specificity	Random Forest	SMOTE	SMOTEENN	2.364	0.029
**Diabetic dataset 2019**					
Accuracy	Random Forest	SMOTE	Borderline-SMOTE	2.987	0.017
ROC AUC	Random Forest	SMOTE	Borderline-SMOTE	2.746	0.022
F1-score	Random Forest	SMOTE	SMOTEENN	3.334	0.010
Precision	Random Forest	SMOTE	SMOTEENN	3.546	0.008
Recall	Random Forest	SMOTE	SMOTEENN	3.776	0.005
Specificity	Random Forest	SMOTE	SMOTEENN	3.012	0.016
**BIT 2019**					
Accuracy	Random Forest	SMOTE	Borderline-SMOTE	2.589	0.032
ROC AUC	Random Forest	SMOTE	Borderline-SMOTE	2.448	0.038
F1-score	Random Forest	SMOTE	SMOTEENN	3.044	0.016
Precision	Random Forest	SMOTE	SMOTEENN	3.667	0.006
Recall	Random Forest	SMOTE	SMOTEENN	3.892	0.005
Specificity	Random Forest	SMOTE	SMOTEENN	2.976	0.017

For the PIMA dataset, the Random Forest classifier, in combination with ADASYN, SMOTE, or Borderline-SMOTE, proved to be the best-performing combination. Random Forest with SMOTE did well in the range of metrics (accuracy, ROC AUC, and F1-acore). The associated *t*-statistics and *p*-values suggest these improvements are statistically significant, as most *p*-values are under 0.05, which might lead us to believe that the performance improvement observed is unlikely due to random chance.

Random Forest and SMOTE synergy achieved the highest AUC score in the case of the Diabetic Dataset 2019. The *t*-tests revealed that this combination was significantly different to others in terms of accuracy and ROC AUC. The consistently < 0.05 *p*-values confirm that Random Forest + SMOTE is the best-performing modeling approach for this dataset.

On the BIT 2019 dataset, in turn, Random Forest + SMOTE showed outperformance. Significant statistical results are demonstrated among all the measuring indices. The fact that very low *p*-values are achieved across experiment types proves this approach's better performance isn't due merely to random variation.

The statistical significance of these results highlights the effectiveness of the Random Forest model, particularly when paired with SMOTE or similar resampling methods, in handling imbalanced datasets. The consistently lower *p*-values across all datasets confirm that these models are not merely outperforming alternatives by chance and are more accurate and reliable. This is due to the synergy between Random Forest's ability to capture complex patterns and SMOTE's technique of generating synthetic samples to balance the dataset, which enhances the model's capacity to generalize and reduce bias toward the majority class. Additionally, SMOTE's introduction of variability in the training data helps mitigate overfitting, further complementing Random Forest's ensemble approach to variance reduction. Consequently, the Random Forest + SMOTE combination is the most statistically significant and practical approach, consistently providing superior predictive accuracy, recall, and other performance metrics across all datasets studied.

### 5.6 Performance analysis across datasets

Our evaluation of the PIMA, Diabetes 2019, and BIT_2019 datasets highlights significant performance distinctions among machine learning models, with Random Forest and XGBoost consistently excelling in accuracy and sensitivity across datasets.

**PIMA Dataset:** Models using imbalance techniques (e.g., ADASYN, SMOTE), especially Random Forest and XGBoost, achieved near-perfect accuracy, F1-score, and ROC-AUC. These models showed strong recall for the minority (diabetic) class while maintaining high specificity, demonstrating robustness with resampled data.

**Diabetes 2019 Dataset:** K-Nearest Neighbors (KNN) and Random Forest, in combination with ADASYN and SMOTE, delivered top sensitivity and specificity, with KNN achieving 97.25% accuracy and a 95.04% F1-score, leveraging local data patterns. Random Forest further improved stability and recall, achieving high ROC-AUC and showcasing its generalization ability in complex data environments.

**BIT_2019 Dataset:** Random Forest and XGBoost, especially with SMOTE and Borderline-SMOTE, reached nearly 98% in ROC-AUC and specificity, effectively addressing imbalance and accurately predicting diabetic cases with minimized false positives.

Overall, the framework proves reliable for diabetes classification across varied datasets, showing strong adaptability to dataset complexity and imbalance levels.

### 5.7 Comparison with the state-of-the-art

In this section, we present a detailed comparison of our proposed methodology against several state-of-the-art techniques from studies published between 2020 and 2024. These studies employed various machine learning methods for diabetes prediction across three datasets: PIMA, Diabetes Dataset 2019, and BIT_2019. To evaluate the effectiveness of these approaches, we compare key metrics including accuracy, precision, recall, F1-score, and specificity (where applicable). It is important to note that in the Tables 7–9, the abbreviation “**NR**” indicates that the corresponding metric was not reported in the referenced studies.

**Table 7:** Performance comparison on PIMA dataset.

**Study**	**Accuracy**	**Precision**	**Recall**	**F1-score**
Stacking (Rajagopal et al., 2022)	0.78	0.72	0.51	0.59
NSGA-II-Stacking (Singh and Singh, 2020)	0.83	**NR**	**NR**	0.88
Morgan-Benita et al. (2022)	0.88	0.88	0.92	0.85
Voting Classifier (XGB + RF) (Kibria et al., 2022)	0.90	0.89	0.88	0.89
SVM (You and Kang, 2020b)	0.71	0.67	0.44	0.53
Saxena et al. (2023)	0.86	0.88	0.79	0.82
XGBoost with ADASYN (Tasin et al., 2023)	0.88	0.82	0.80	0.81
Random Forest (Chang et al., 2023)	0.80	0.89	**NR**	0.85
Stacking 2A (Daza et al., 2024)	0.88	0.88	0.86	0.88
Stacking 1A (Daza et al., 2024)	0.91	0.91	0.91	0.91
Voting (Chowdhury et al., 2024)	0.728	0.625	0.68	**NR**
**The proposed system**	**1.0**	**1.0**	**1.0**	**1.0**

NR signifies that the corresponding metric was not reported in the referenced studies. The bolded results represent the outcomes of the proposed system, which achieved state-of-the-art performance across all three datasets.

As shown in Table 7, our proposed methodology demonstrates a substantial improvement over existing approaches on the **PIMA dataset**. By leveraging Random Forest in combination with SMOTE, our method achieves perfect scores across all metrics, including accuracy (1.0), precision, recall, and F1-score. In contrast, the best-performing prior study, Stacking 1A (Logistic regression), attained an accuracy of 0.91. This significant enhancement can be attributed to the effective handling of class imbalances by SMOTE, which prevents the Random Forest model from favoring majority classes, thereby improving its generalization capability.

As indicated in Table 8, our methodology achieves superior performance on the **Diabetes Dataset 2019**, recording an accuracy of 0.98. This surpasses the Decision Tree method, which achieved an accuracy of 0.95 and was the closest competitor. Our approach demonstrates its effectiveness in addressing the complexities of this dataset, outperforming traditional methods such as Logistic Regression and SVM, which exhibited comparatively lower performance. The synergy between the robust Random Forest model and the balanced datasets generated through SMOTE contributes significantly to this improvement.

**Table 8:** Performance comparison on diabetes dataset 2019.

**Study**	**Accuracy**	**Precision**	**Recall**	**F1-score**
Linear regression (Uddin et al., 2024)	0.89	**NR**	**NR**	**NR**
Logistic regression (Uddin et al., 2024)	0.89	**NR**	**NR**	**NR**
SVM (Uddin et al., 2024)	0.93	**NR**	**NR**	**NR**
Naive Bayes (Uddin et al., 2024)	0.85	**NR**	**NR**	**NR**
Decision Tree (Uddin et al., 2024)	0.95	**NR**	**NR**	**NR**
KNN (Uddin et al., 2024)	0.92	NR	NR	NR
**The proposed system**	**0.98**	**0.97**	**0.96**	**0.97**

NR signifies that the corresponding metric was not reported in the referenced studies. The bolded results represent the outcomes of the proposed system, which achieved state-of-the-art performance across all three datasets.

As reflected in Table 9, our methodology achieves the highest performance on the **BIT_2019 dataset**, with an accuracy of 0.98, an F1-score of 0.96, and a specificity of 0.99. These results surpass advanced techniques such as the ResNet-50 model, which achieved an accuracy of 0.91, and the BPNN + BatchNorm approach, which reported slightly lower specificity and F1-score. The ability of our method to outperform even sophisticated deep learning models highlights the strength of ensemble methods like Random Forest, particularly when combined with effective data resampling techniques. These findings reinforce the generalization and robustness of our approach across diverse datasets.

**Table 9:** Performance comparison on BIT_2019 dataset.

**Study**	**Accuracy**	**Precision**	**Recall**	**F1-score**	**Specificity**
XGBoost (Zhang et al., 2024)	0.9245	**NR**	**NR**	0.9524	0.9062
K-Means Clustering (Zhang et al., 2024)	0.7264	**NR**	**NR**	0.4762	0.8906
SOM (Zhang et al., 2024)	0.6698	**NR**	**NR**	0.5714	0.7344
ResNet-14 (Zhang et al., 2024)	0.9245	**NR**	**NR**	0.9524	0.9063
ResNet-50 (Zhang et al., 2024)	0.9151	**NR**	**NR**	0.9286	0.9062
BPNN + BatchNorm (Zhang et al., 2024)	0.9528	**NR**	**NR**	0.95	0.9219
**The proposed system**	**0.98**	**0.96**	**0.97**	**0.96**	**0.99**

NR signifies that the corresponding metric was not reported in the referenced studies. The bolded results represent the outcomes of the proposed system, which achieved state-of-the-art performance across all three datasets.

These results underscore the potential of our methodology for practical applications in clinical settings, where early and accurate detection can significantly improve patient outcomes. Future work will explore the application of this approach to additional datasets and investigate its integration into real-time diagnostic tools.

## 6 Discussion

### 6.1 Influence of imbalance handling techniques on model sensitivity

The findings in this study reveal the significant impact of using resampling techniques, such as ADASYN, SMOTE, and Borderline-SMOTE, on improving model sensitivity, particularly for predicting minority classes in imbalanced datasets. These techniques play a critical role in medical data analysis, where the goal is to maximize accuracy and ensure that minority cases—in this case, diabetic patients—are accurately identified.

Resampling methods generate synthetic samples for the minority class, which helps balance the dataset and mitigate the model's tendency to favor the majority class. For example, in both the PIMA and BIT_2019 datasets, applying ADASYN enabled Random Forest to reach 100% recall, ensuring that all diabetic cases were correctly identified. This increase in recall, without compromising specificity, is essential in healthcare settings, where the cost of a false negative (missed diagnosis) is high. Identifying diabetic patients early can lead to timely interventions, which may prevent the progression of the disease and reduce the risk of severe complications.

### 6.2 Model performance with resampling techniques

The ensemble models—Random Forest and XGBoost—consistently outperformed other models across all datasets, especially when coupled with resampling techniques. Random Forest and XGBoost are known for their robustness and ability to handle complex data patterns, which is further enhanced by applying ADASYN and SMOTE. By creating synthetic samples, these techniques allow the models to learn a more balanced decision boundary, resulting in higher sensitivity and specificity.

Notably, the high F1-scores and ROC-AUC values achieved by Random Forest and XGBoost in the Diabetes 2019 dataset suggest that these models are not only effective in classifying diabetic patients but also resistant to overfitting on the majority class. The F1-score reflects the harmonic mean of precision and recall, emphasizing the model's capacity to make reliable predictions across classes. Similarly, high ROC-AUC values indicate the model's strong ability to differentiate between diabetic and non-diabetic cases, which is critical for real-world applications where imbalanced data is common.

### 6.3 Clinical implications of high specificity and ROC-AUC

High specificity and ROC-AUC values in this study have important implications for diabetes prediction in clinical practice. Specificity reflects the model's ability to correctly identify non-diabetic cases, thus minimizing the likelihood of overdiagnosis, which can lead to unnecessary anxiety and medical interventions. In clinical contexts, a high specificity rate ensures that patients who are not at risk are correctly identified, reducing the burden on healthcare resources.

ROC-AUC values across the three datasets consistently approached or exceeded 0.95, particularly for the Random Forest and XGBoost models when paired with SMOTE or Borderline-SMOTE. High ROC-AUC values in imbalanced datasets confirm that the models are accurate and unbiased toward any particular class. In practical terms, this robustness means that the model can be deployed in various settings, including remote healthcare environments or as part of telemedicine solutions, where accurate early screening can significantly improve patient outcomes.

### 6.4 Generalizability of the proposed framework

The performance of the proposed framework across different datasets suggests strong potential for generalizability. Each dataset—PIMA, Diabetes 2019, and BIT_2019—varies in its feature composition and level of class imbalance, yet the framework consistently provided high sensitivity, specificity, and F1-scores. This adaptability suggests that the framework could be applied to other chronic disease datasets or modified for similar imbalanced data scenarios in healthcare applications.

## 7 Conclusion and future work

This study evaluated twelve machine learning models combined with five resampling techniques—SMOTE, ADASYN, Borderline-SMOTE, Random Under Sampling, and SMOTEENN—across three datasets (PIMA, Diabetes Dataset 2019, and BIT_2019) for diabetes prediction. Among the models, Random Forest with SMOTE consistently achieved the highest accuracy, F1-score, and ROC-AUC, demonstrating robust performance in handling imbalanced data and accurately predicting minority cases. This combination underscores the scientific contribution of pairing ensemble models with effective resampling techniques to improve minority class prediction in imbalanced datasets. The results show high sensitivity, specificity, and ROC-AUC, making the approach clinically relevant by reducing risks of overdiagnosis and missed diagnoses and offering reliable, actionable insights for healthcare providers.

While these findings are promising, future work could extend this framework's evaluation to larger and more complex medical datasets to explore its broader applicability across diverse health conditions. In addition, applying advanced deep learning models, particularly on datasets with substantial sample sizes, may further enhance scalability and predictive power. Testing this approach in real-time clinical settings could provide critical insights into processing speed, data privacy, and interpretability, thus supporting the transition of machine learning models from research to practical clinical applications and contributing to more data-driven, precise healthcare interventions.

## Data Availability

The data supporting this study are available from the corresponding author upon reasonable request.
